# Stereoselective Four-Component Synthesis of Functionalized 2,3-Dihydro-4-Nitropyrroles

**DOI:** 10.3389/fchem.2019.00810

**Published:** 2019-11-26

**Authors:** Dong Wang, Xinyue Ma, Linru Dong, Hairong Feng, Peng Yu, Laurent Désaubry

**Affiliations:** ^1^Sino-French Joint Lab of Food Nutrition/Safety and Medicinal Chemistry, College of Biotechnology, Tianjin University of Science and Technology, Tianjin, China; ^2^Laboratory of Medicinal Chemistry and Cardio-oncology, FRE2033, CNRS, Institut Le Bel, Strasbourg, France

**Keywords:** multicomponent reaction, ketoamide, heterocycles, nitro, dihydropyrroles, cascade reaction, 1, 5-dipolar cyclization

## Abstract

We report a metal-free and stereoselective four-component reaction between α-ketoamides, amines, aromatic aldehydes and β-nitroalkenes or β-pivaloxy-nitroalkanes to obtain 2,3-dihydro-4-nitropyrroles functionalized in every position. The heterocycles accessible using this reaction may have utility in the synthesis of pharmacologically active compounds.

## Introduction

**Scheme 1:**
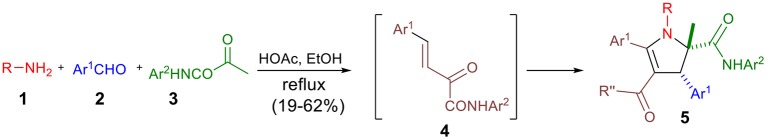
Catalyst-free three-component synthesis of 2,3-dihydropyrroles **5**.

The formation of novel chemical scaffold represents a critical step in drug discovery to generate original drug candidates (Kim et al., 2014). In this quest, multicomponent reactions are very attractive as they provide a wealth of complex products in only one step (Armstrong et al., 1996; Colombo and Peretto, 2008; Magedov et al., 2008; Domling et al., 2012; Bonne et al., 2013; Ruijter and Orru, 2013; Cores et al., 2014; Zarganes-Tzitzikas and Domling, 2014; Zhu et al., 2014; Herrera and Marques-López, 2015). Recently, our group reported a novel multicomponent condensation of amines, aromatic aldehydes and α-ketoamides to provide fully substituted 2,3-dihydropyrroles **5** in an excellent highly stereoselective, atom economic, and eco-friendly fashion (Scheme 1) (Wang et al., 2018).

The drug-like character of 2,3-dihydropyrroles prompted us to examine their pharmacological potential, which led to the discovery of potent α-glucosidase inhibitors, illustrating the utility of this novel methodology in medicinal chemistry (Wang et al., 2018).

**Scheme 2:**
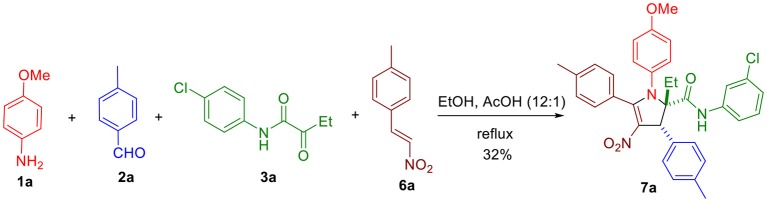
Three-component synthesis of 2,3-dihydro-4-nitropyrrole **7a**.

To expand the scope of this reaction and generate 2,3-dihydropyrroles with new types of substituents, we considered using β-nitrostyrenes **6** susceptible to react similarly to the intermediate enone **4** (Scheme 2).

## Results and Discussion

**Figure 1:**
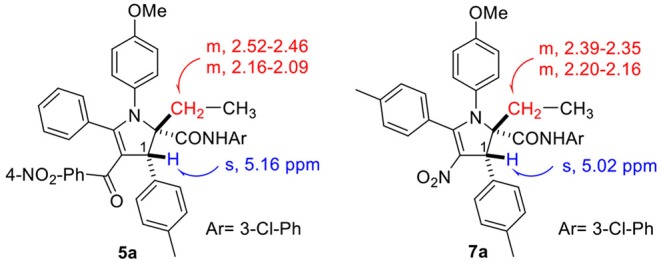
NMR features of 2,3-dihydropyrroles.

**Scheme 3:**
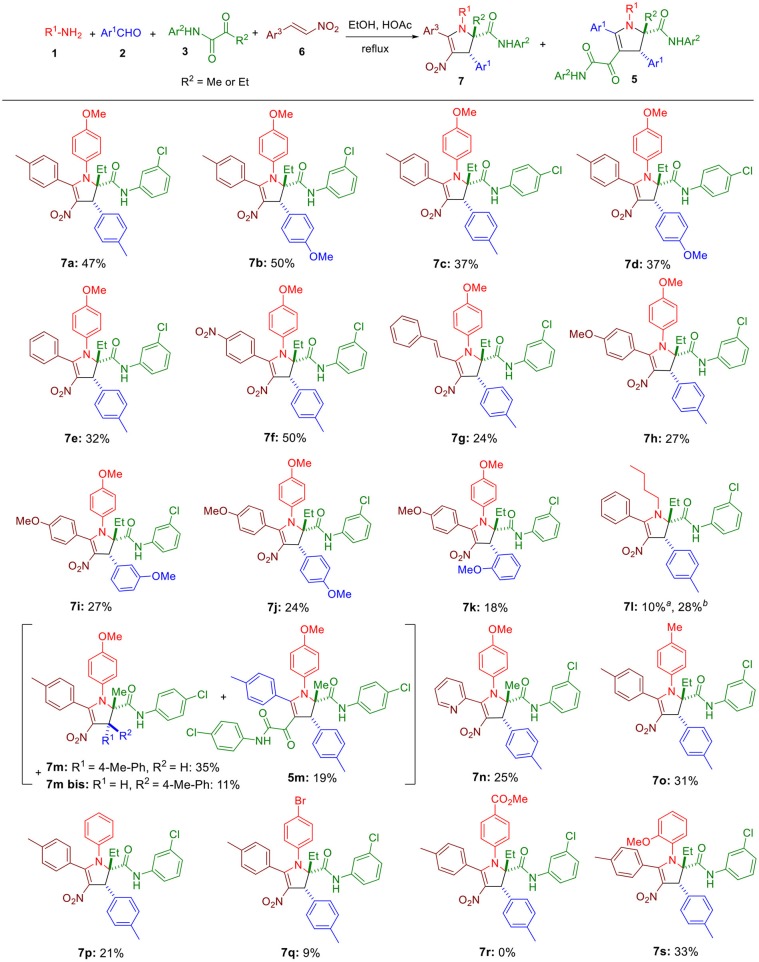
Substrate scope for the four-component reaction. Unless otherwise noted, all reactions were conducted at 0.05 M concentration with an equimolar concentration of the four reagents in EtOH-AcOH (12:1) under reflux conditions, and an extra eq. of both **1** and **6** were added to the reaction medium after 4 h. All reactions were conducted for 10 h. ^*a*^Reaction was done without any acetic acid. ^*b*^Reaction was conducted with an equimolar concentration of the four reagents in EtOH (0.025 M) under reflux conditions.

Using the optimized conditions identified in our previous study (10 h reflux in EtOH/AcOH 12:1, entry 1, Table 1), we were pleased to observe in our first attempt the generation of the expected 4-nitro-2,3-dihydropyrrole **7a** in 32% yield, which was purified by flash chromatography. The singlet peak at 5.02 ppm in the proton NMR clearly reveals the existence of H^1^ (Figure 1, both at benzylic and allylic position), which is coherent with the one observed for compound **5a** disclosed in our previous article (Wang et al., 2018). Moreover, two separate multiplets (at about 2.37 and 2.18 ppm) corresponding to CH_2_ group (Scheme 3) indicates they are diastereotopic protons, further confirming the structure (detailed experimental procedures and experimental data are disclosed in the Supplementary Material).

**Table 1:** Optimization of the synthesis of 2,3-dihydro-4-nitropyrrole **7a**.^*^

**Entry**	**Mol. ratio**	**[M]**	**Yield (%)**
	**(1a:2a:3a:6a)**		
1	1:1:1:1	0.025	32
2*^a^*	1:1:1:1	0.025	0
3	1:1:1:1	0.05	30
4	1:1:1:1	0.1	35
5*^b^*	1:1:1:1	0.05	27
6	2:2:1:1	0.025	38
7	2:2:1:1	0.05	20
8	2:1:1:1	0.025	19
9	4:4:1:1	0.025	15
10	2:1:1:2	0.025	32
11*^c^*	2:1:1:2	0.025	31
12*^c^*	2:1:1:2	0.050	47

* *Unless otherwise noted, all reactions were conducted with **3a** (80 mg, 1.0 eq) in EtOH-HOAc (12:1) under reflux conditions for 10 h*.

a *Reaction run in a sealed tube at 110°C*.

b *Reaction run in EtOH-HOAc (6:1)*.

c *Reaction run with **1a**:**2a**:**3a**:**6a** = 1:1:1:1 at the beginning, after 4 h stirring at reflux temperature, extra **1a** (1 eq) and **6a** (1 eq) were added to the reaction mixture*.

Since homopyruvic amide **3a** was not completely consumed, other reaction conditions were examined (Table 1). Heating the reaction to higher temperature did not yield the expected adduct (entry 2), probably due to thermal decomposition. More concentrated reaction media gave similar yields (entries 3 and 4). Similarly, adding more acetic acid to the reaction media did not offer improved yield either (entry 5). Doubling the amount of **1a** and **2a** slightly increased the yield to 38% (entry 6). However, changing the concentration or the molar ratio further did not lead to better result (entries 7–9). Since both amine **1a** and β-nitrostyrene **6a** were consumed after running the reaction for 4 h, we doubled their amount at the beginning of the reaction and also 4 h later, but it had little effect on the yield (entries 10 and 11). Eventually, performing this latter condition in a more concentrated condition increased the yield to 47% (entry 12).

Next, the substrate scope was explored. Replacing tolualdehyde by anisaldehyde did not significantly modify the yield (**7b**, Scheme 3). Moving the position of the chlorine in the ketoamide lowered the yield by about 10% (**7c**, **7d**). Switching the methyl in the β-nitrostyrenes **6** to a nitro (**7f**) was well tolerated, but replacing it with a hydrogen (**7e**), extending the conjugation by adding an extra double bond (**7g**) or lowering the electrophilicity by introducing a 4-methoxy in the phenyl moiety (**7h**-**7k**) reduced the yields to 32% and 24–27%. We found that the replacement of anisidine by *n*-butylamine yielded the expected product **7l** (28%) when acetic acid was absent in the medium, confirmining the suitability of aliphatic amines for this reaction. Using a pyruvic amide **3** (*R*^2^ = Me instead of Et), we observed the formation of the expected adduct **7m** accompanied by its diastereomeric isomer **7m bis** and the ketoamide **5m** generated by the α,β-enones resulting from the condensation of aldehydes **2** and the pyruvic amide. Using a more electrophilic β-nitrostyrene such as 2-(2-nitrovinyl)pyridine yielded only the expected adduct **7n**. Importantly, decreasing the basicity of the aniline **1** was highly detrimental (**7o**-**7r**), but shifting the methoxy of anisidine to the ortho position was well tolerated.

**Scheme 4:**
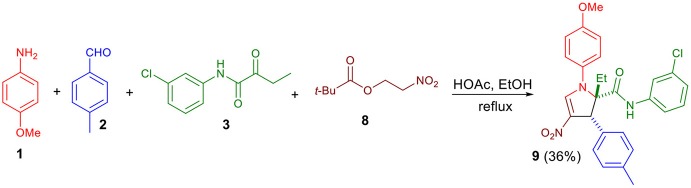
Condensation reactions with β-pivaloxy-nitrsoalkane **8**.

Next, we examined whether β-pivaloxy-nitroalkane could be used as a reactant (Scheme 4). Condensation with **8** afforded the expected adduct (**9**) unsubstituted at the C5-position, with yield similar to those observed with β-nitrostyrenes and aromatic aldehydes.

**Scheme 5:**
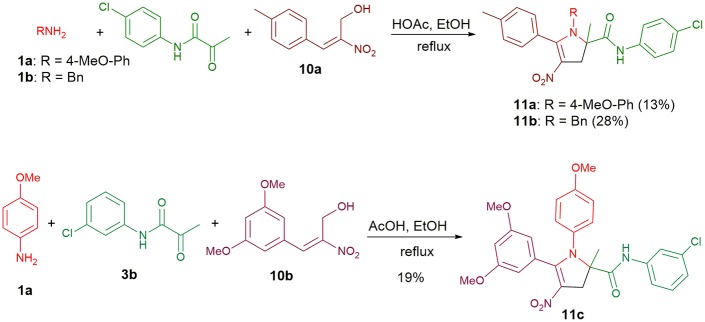
Condensations with allylic alcohols **10a** and **10b**.

Furthermore, allylic alcohols **10a** and **10b** were also viable substrates that allowed the synthesis of 2,3-dihydro-4-nitropyrroles **11a-c** unsubstituted at the C3-position (Scheme 5). Having the adduct **11c** in hand, we examined whether this nitrostyrenic derivative could undergo a reductive cyclization using TiCl_3_ as a reductant (Tong et al., 2015). So far as we know, this approach to synthesize functionalized indoles from *o*-nitrostyrenes has never been reported with the nitro group appended to a vinyl moiety rather than an arene.

**Scheme 6:**
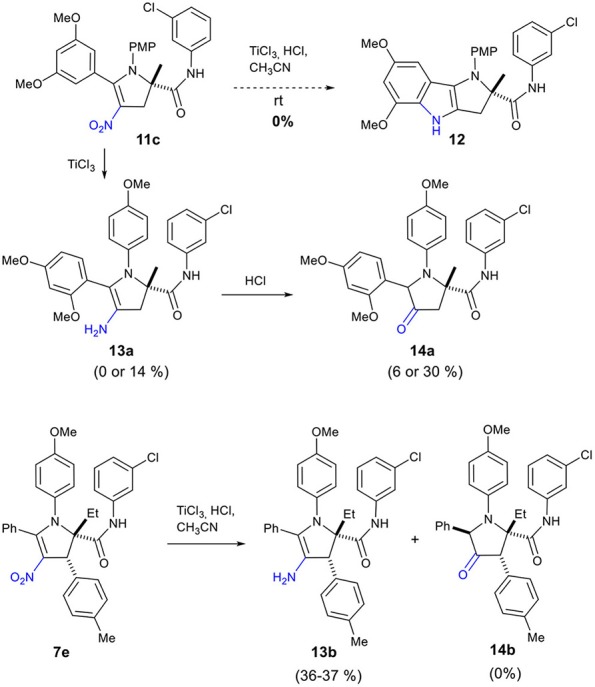
Reductive transformation of 2,3-dihydro-4-nitropyrrole **11c** to enamine **13a** and ketone **14a** and conversion of **7e** to **13b**.

Our attempt to prepare the tetrahydropyrrolo[3,2-*b*]indole **12** from the adduct **11c** by treatment with TiCl_3_ did not deliver this cyclized compound **12** (Scheme 6). Instead, we observed the formation of enamine **13a** or ketone **14a** depending on the reaction conditions (Table 2). Reduction of the nitro **11c** by TiCl_3_ gave the enamine **13a** as the major product in 14% yield, when the reaction was performed for 1.5 h at a 0.2 M concentration (entry 1, Table 2). Under more concentrated reaction conditions and with a reaction time extended to 14 h, only the pyrrolidinone **14a**, resulting from the hydrolysis of **13a**, was obtained (entry 2).

**Table 2:** Effect of reaction conditions on the reduction of **11c** and **7e**.

**Entry**	**Substrate**	**Condition**	**13, yield**	**14, yield**
1	**11c**	0.2 M, 1.5 h	**13a**, 14%	**14a**, 6%
2		0.4 M, 14 h	**13a**, 0%	**14a**, 30%
3	**7e**	0.2 M, 2.5 h	**13b**, 37%	**14b**, 0%
4		0.4 M, 14 h	**13b**, 36%	**14b**, 0%

Surprisingly, application of this reaction to dihydro-4-nitropyrrole **7e** provided dihydro-4-aminopyrrole **13b** as the sole product in both conditions (entries 3 and 4), possibly due to the enhanced steric hindrance which prohibits the hydrolysis of the enamine. This sequence is noteworthy, as 1,2,4,5,5-pentasubstituted 3-amino-4,5-dihydro-1H-pyrroles have been scarcely reported, indicating a high potential to generate intellectual property in medicinal chemistry programs

**Scheme 7:**
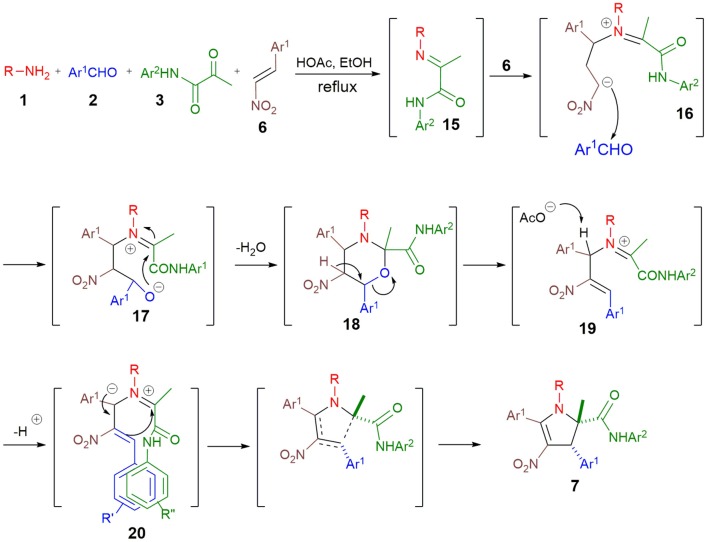
Four-component synthesis of 2,3-dihydropyrroles **7** and proposed mechanism.

On the basis of investigations previously reported by our group (Wang et al., 2018), we proposed that mechanism of this reaction involves the conjugate addition of imine **15** to nitrostyrene **6** to produce intermediate **16** that reacts with aldehyde **2**, leading to 3-oxazine **18** (Scheme 7). Successive dehydration and deprotonation then generate the azomethine ylide **20** that undergoes an intramolecular 1,5-dipolar cycloaddition (Taylor, 1979) to afford the 2,3-dihydropyrrole **7**. The stabilization of the intermediate **20** by π-stacking between the Ar^1^ and Ar^2^ probably account for the observed diastereospecificity of this reaction.

## Conclusion

We have developed a multicomponent reaction that uses α-ketoamides, amines, aromatic aldehydes and β-nitrostyrenes or β-pivaloxy-nitroalkanes to deliver functionalized 2,3-dihydro-4-nitropyrroles in moderate yields (9–50%), however considering that this reaction generates four new bonds, it is still effective (55–84% average yield per bond formation). Combining our multicomponent reaction with a TiCl_3_-induced reduction gives access to novel polysubstituted dihydro-1H-pyrroles.

## Data Availability Statement

The raw data supporting the conclusions of this manuscript will be made available by the authors, without undue reservation, to any qualified researcher.

## Author Contributions

LDé and DW were responsible for designing the experiments. XM, LDo, and HF performed the experimentations. LDé, DW, and PY analyzed the results and wrote the publication.

### Conflict of Interest

The authors declare that the research was conducted in the absence of any commercial or financial relationships that could be construed as a potential conflict of interest.
