# Supplementary material for: Stereoselective Four-Component Synthesis of Functionalized 2,3-Dihydro-4-Nitropyrroles
Source: Front Chem. 2019 Nov 26;7:810. doi: 10.3389/fchem.2019.00810 (PMC6988783; doi:10.3389/fchem.2019.00810)
Supplement: Supplementary file 1 [file Presentation_1.pdf]

*Supplementary Material*

# Stereoselective Four-Component Synthesis of Functionalized 2,3-Dihydro-4-Nitropyrroles

**Dong Wang,[1]* Xinyue Ma,[1] Linru Dong,[1] Hairong Feng,[1] Peng Yu [1], and Laurent Désaubry[1,2]***

[1]Sino-French Joint Lab of Food Nutrition/Safety and Medicinal Chemistry , College of Biotechnology, Tianjin University of Science and Technology, Tianjin 300457, China
[2]Laboratory of Medicinal Chemistry and Cardio-oncology, FRE2033, CNRS, Institut Le Bel , 4 rue Blaise Pascal , 67008 Strasbourg, France

**Correspondence:**
wangdong@tust.edu.cn, desaubry@unistra.fr

## Table of Contents

1. General Experimental ........................................ 2
2. Preparation and characterization for the products ........................................ 3
3. NOESY for **7m**, **7m-bis** and **14a** ........................................ 17
4. Reference ........................................ 19
5. Spectra Data ........................................ 19

**1. General Experimental**

The preparation experiments were performed under air or an argon atmosphere in oven dried glassware. Solvents used as reaction media were distilled immediately before use: THF was distilled from Na/benzophenone ketyl, DCM and DCE were distilled from calcium hydride, DMF was obtained from vacuum distillation, EtOH was distilled from Mg and $I_2$. All reagents were purchased at the highest commercial quality and used without further purification. Reactions were monitored by thin layer chromatography (TLC) using ultra violet light (UV) as the visualizing agent. Nuclear magnetic resonance spectra (NMR) were recorded on Bruker AV-400 instruments and were calibrated using residual undeuterated solvent as an internal reference ($^1$H NMR: $CHC1_3$ 7.26 ppm, $^{13}$C NMR: $CHC1_3$ 77.16 ppm). High resolution mass spectra (HRMS) were recorded on a hybrid IT-TOF mass spectrometer (Shimadzu LCMS-IT-TOF, Kyoto, Japan). The following abbreviations were used to indicate multiplicities: s = singlet, d = doublet, t = triplet, q = quartet, quin = quintet, sex = sextet, sep = septet, dd = doublet of doublets, dt = doublet of triplets, ddd = doublet of doublet of doublets, ddt = doublet of doublet of triplets, m = multiplet).

## General Procedure A: four-component synthesis of 2,3-dihydro-4-nitropyrroles (7)

A solution of amine (1.0 equiv), aldehyde (1.0 equiv), pyruvic amide (1.0 equiv), β-nitroalkene (1.0 equiv) and acetic acid ($V_{EtOH}$ : $V_{AcOH}$ = 12:1) in anhydrous EtOH (amine concentration: 0.05 mol/L) was stirred under reflux for 4 h, then extra amine (1.0 equiv) and β-nitroalkene (1.0 equiv) was added to the reaction mixture under reflux and stirring was continued for another 6 h. After cooled down to r.t., ethanol was removed under vacuum, and the residue was diluted with EtOAc and water. After separation, the aqueous phase was extracted two more times with EtOAc. The combined organic phases were successively washed with saturated aqueous $KHSO_4$, saturated aqueous $NaHCO_3$, and brine, dried over $MgSO_4$, concentrated *in vacuo* and purified using gradient elution flash column chromatography on silica gel with petroleum ether/ethyl acetate (5:1~3:1) to give the dihydropyrrole products.

## General Procedure B: three-component synthesis of 2,3-dihydro-4-nitropyrroles (11)

A solution of amine (1.0 equiv), pyruvic amide (1.0 equiv), allylic alcohol (1.0 equiv) and acetic acid ($V_{EtOH}$ : $V_{AcOH}$ = 12:1) in anhydrous EtOH (amine concentration: 0.025 mol/L) was stirred under reflux for 8 h. After cooled down to r.t., ethanol was removed under vacuum, and the residue was diluted with EtOAc and water. After separation, the aqueous phase was extracted two more times

with EtOAc. The combined organic phases were successively washed with saturated aqueous $KHSO_4$, saturated aqueous $NaHCO_3$, and brine, dried over $MgSO_4$, concentrated *in vacuo* and purified using gradient elution flash column chromatography on silica gel with petroleum ether/ethyl acetate (5:1~3:1) to give the dihydropyrrole products.

## 2. Preparation and characterization for the products

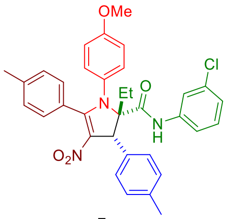


**7a**

**(2RS,3RS)-N-(3-chlorophenyl)-2-ethyl-1-(4-methoxyphenyl)-4-nitro-3,5-di-p-tolyl-2,3-dihydro-1H-pyrrole-2-carboxamide (7a)**

Using N-(3-chlorophenyl)-2-oxobutanamide (80 mg, 0.38 mmol), in accordance with General Procedure A, the title compound was obtained (103 mg, 47% yield) as a yellow solid (m.p. 101.3-103.9°C). $^1H$ NMR (400 MHz, $CDCl_3$) δ 7.24 (d, $J$ = 1.6 Hz, 1H), 7.12 (q, $J$ = 8.4 Hz, 4H), 7.04 (t, $J$ = 8.0 Hz, 3H), 6.98 (d, $J$ = 12.4 Hz, 2H),6.88 (d, $J$ = 9.2 Hz, 2H), 6.68 (s, 1H), 6.61 (d, $J$ = 8.8 Hz, 3H), 5.00 (s, 1H), 3.67 (s, 3H), 2.32 - 2.36 (m, 1H), 2.31 (s, 3H), 2.22 (s, 3H), 2.14 - 2.20 (m, 1H), 1.22 (t, $J$ = 6.4 Hz, 3H). $^{13}C$ NMR (400 MHz, $CDCl_3$) δ 167.1, 160.9, 158.3, 140.6, 138.0, 137.4, 134.3, 133.8, 130.5, 129.7, 129.6, 129.09, 129.06, 127.9, 126.2, 125.1, 124.4, 121.3, 119.1, 114.3, 81.4, 55.5, 54.2, 28.9, 21.7, 21.1, 8.2. HRMS (+ESI-TOF) *m/z*: $[M+H]^+$ Calcd for $C_{34}H_{33}N_3O_4Cl$: 582.2154; Found 582.2150.

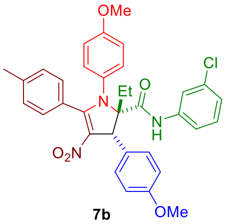


**7b**

**(2RS,3RS)-N-(3-chlorophenyl)-2-ethyl-1,3-bis(4-methoxyphenyl)-4-nitro-5-(p-tolyl)-2,3-dihydro-1H-pyrrole-2-carboxamide (7b)**

Using N-(3-chlorophenyl)-2-oxobutanamide (80 mg, 0.38 mmol), in accordance with General Procedure A, the title compound was obtained (113 mg, 50% yield) as a yellow solid (m.p. 103.7-106.2°C). $^1H$ NMR (400 MHz, $CDCl_3$) δ 7.34 (d, $J$ = 8.8 Hz, 2H), 7.16 (d, $J$ = 8.0 Hz, 2H), 7.07-7.05

(m, 3H), 7.04-7.00 (m, 1H), 6.90-6.88 (m, 3H), 6.83 (d, $J$ = 8.8 Hz, 2H), 6.69-6.64 (m, 4H), 5.02 (s, 1H), 3.80 (s, 3H), 3.70 (s, 3H), 2.39-2.33 (m, 1H), 2.31 (s, 3H), 2.24-2.20 (m, 1H), 1.25 (t, $J$ = 7.2 Hz, 3H); $^{13}$C NMR (100 MHz, $CDCl_3$) δ 167.1, 161.0, 160.9, 158.3, 137.9, 137.5, 134.2, 133.9, 131.6, 131.5, 130.7, 129.6, 129.1, 127.9, 125.1, 124.1, 121.4, 121.0, 119.2, 114.4, 113.8, 81.3, 55.5, 55.4, 54.2, 29.1, 21.1, 8.2. HRMS (-ESI-TOF) m/z: $[M - H]^-$ calcd for $C_{34}H_{31}N_3O_5Cl$ 596.1958; found 596.1962.

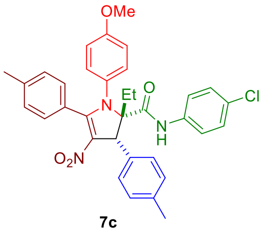


7c

**(2RS,3RS)-N-(4-chlorophenyl)-2-ethyl-1-(4-methoxyphenyl)-4-nitro-3,5-di-p-tolyl-2,3-dihydro-1H-pyrrole-2-carboxamide (7c)**

Using N-(4-chlorophenyl)-2-oxobutanamide (80 mg, 0.38 mmol), in accordance with General Procedure A, the title compound was obtained (82 mg, 37% yield) as a yellow solid (m.p. 86.0-89.2°C). $^1$H NMR (400 MHz, $CDCl_3$) δ 7.28 (s, 1H), 7.17-7.08 (m, 6H), 7.04-6.99 (m, 3H), 6.89 (d, $J$ = 8.8 Hz, 2H), 6.69 (d, $J$ = 8.4 Hz, 2H), 6.63 (d, $J$ = 8.8 Hz, 2H), 5.02 (s, 1H), 3.69 (s, 3H), 2.39-2.35 (m, 1H), 2.33 (s, 3H), 2.22 (s, 3H), 2.20-2.16 (m, 1H), 1.24 (t, $J$ = 7.2 Hz, 3H); $^{13}$C NMR (100 MHz, $CDCl_3$) δ 167.0, 160.8, 158.3, 140.6, 137.8, 134.9, 133.9, 130.6, 130.1, 129.6, 129.1, 129.0, 128.7, 127.9, 126.2, 124.5, 122.1, 114.3, 81.4, 55.5, 54.2, 28.9, 21.7, 21.1, 8.2. HRMS (-ESI-TOF) m/z: $[M - H]^-$ calcd for $C_{34}H_{31}N_3O_4Cl$ 580.2009; found 580.2009.

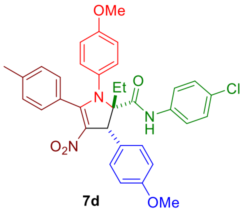


7d

**(2RS,3RS)-N-(4-chlorophenyl)-2-ethyl-1,3-bis(4-methoxyphenyl)-4-nitro-5-(p-tolyl)-2,3-dihydro-1H-pyrrole-2-carboxamide (7d)**

Using N-(4-chlorophenyl)-2-oxobutanamide (80 mg, 0.38 mmol), in accordance with General Procedure A, the title compound was obtained (83 mg, 37% yield) as a yellow solid (m.p. 108.2-111.4°C). $^1$H NMR (400 MHz, $CD_3OD$) δ 7.37 (d, $J$ = 8.0 Hz, 2H), 7.22 (d, $J$ = 8.0 Hz, 2H), 7.12-7.05 (m, 6H), 6.85 (d, $J$ = 9.2 Hz, 2H), 6.74-6.70 (m, 4H), 4.97 (s, 1H), 3.77 (s, 3H), 3.68 (s, 3H), 2.54-2.49 (m, 1H), 2.27 (s, 3H), 2.24-2.20 (m, 1H), 1.31-1.27 (m, 3H); $^{13}$C NMR (100 MHz,

CD$_3$OD) δ 168.9, 165.2, 162.4, 160.2, 138.4, 136.8, 135.5, 132.7, 131.8, 131.3, 129.9, 129.7, 129.1, 125.3, 124.8, 122.9, 115.2, 114.9, 114.4, 83.4, 55.83, 55.76, 55.2, 29.0, 21.1, 8.5. HRMS (+ESI-TOF) m/z: $[M + H]^+$ calcd for $C_{34}H_{33}N_3O_5Cl$ 598.2103; found 598.2130.

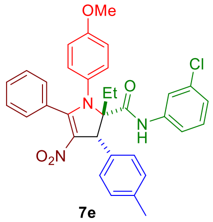


7e

**(2RS,3RS)-N-(3-chlorophenyl)-2-ethyl-1-(4-methoxyphenyl)-4-nitro-5-phenyl-3-(p-tolyl)-2,3-dihydro-1H-pyrrole-2-carboxamide (7e)**

Using N-(3-chlorophenyl)-2-oxobutanamide (80 mg, 0.38 mmol), in accordance with General Procedure A, in accordance with General Procedure A, the title compound was obtained (69 mg, 32% yield) as a yellow solid (m.p. 102.4-105.9°C). $^1$H NMR (400 MHz, CD$_3$OD) δ 7.36-7.26 (m, 6H), 7.22-7.20 (m, 1H), 7.15 (d, $J$ = 8.0 Hz, 2H), 7.11-7.08 (m, 3H), 7.03-7.01 (m, 1H), 6.78 (t, $J$ = 1.6 Hz, 1H), 6.72 (t, $J$ = 8.8 Hz, 3H), 5.02 (s, 1H), 3.69 (s, 3H), 2.60-2.51 (m, 1H), 2.32 (s, 3H), 2.27-2.22 (m, 1H), 1.34-1.29 (m, 3H); $^{13}$C NMR (100 MHz, CD$_3$OD) δ 168.8, 165.6, 160.3, 141.4, 139.3, 138.6, 134.7, 131.5, 130.5, 129.7, 129.3, 128.6, 128.3, 126.1, 124.9, 123.8, 121.9, 114.8, 83.7, 55.8, 55.5, 28.9, 21.5, 8.6. HRMS (+ESI-TOF) m/z: $[M + H]^+$ calcd for $C_{33}H_{31}N_3O_4Cl$ 568.1998; found 568.2003.

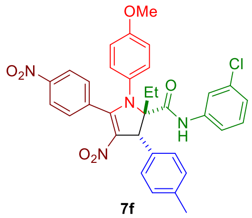


7f

**(2RS,3RS)-N-(3-chlorophenyl)-2-ethyl-1-(4-methoxyphenyl)-4-nitro-5-(4-nitrophenyl)-3-(p-tolyl)-2,3-dihydro-1H-pyrrole-2-carboxamide (7f)**

Using N-(3-chlorophenyl)-2-oxobutanamide (80 mg, 0.38 mmol), in accordance with General Procedure A, the title compound was obtained (116 mg, 50% yield) as a yellow solid (m.p. 120.6-124.0°C). $^1$H NMR (400 MHz, CDCl$_3$) δ 8.01 (d, $J$ = 8.4 Hz, 2H), 7.48-7.43 (m, 3H), 7.26-7.25 (m, 1H), 7.13 (d, $J$ = 8.0 Hz, 2H), 7.06-6.99 (m, 2H), 6.91 (d, $J$ = 8.8 Hz, 2H), 6.83 (s, 1H), 6.67-6.64 (m, 3H), 5.11 (s, 1H), 3.70 (s, 3H), 2.40-2.34 (m, 4H), 2.20-2.12 (m, 1H), 1.28-1.22 (m, 3H); $^{13}$C NMR (100 MHz, CD$_3$OD) δ 168.2, 166.1, 160.5, 148.7, 146.5, 141.6, 139.2, 134.9, 131.6, 131.1, 130.6, 129.7, 127.9, 126.2, 124.1, 123.8, 123.0, 121.2, 114.9, 83.7, 55.8, 55.2, 28.6, 21.5, 8.5. HRMS

(+ESI-TOF) m/z: $[M + H]^+$ calcd for $C_{33}H_{30}N_4O_6Cl$ 613.1848; found 613.1836.

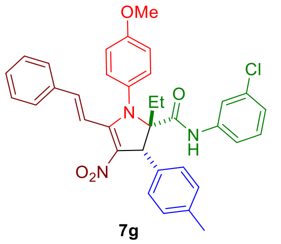

**7g**

**(2RS,3RS)-N-(3-chlorophenyl)-2-ethyl-1-(4-methoxyphenyl)-4-nitro-5-((E)-styryl)-3-(p-tolyl)-2,3-dihydro-1H-pyrrole-2-carboxamide (7g)**

Using N-(3-chlorophenyl)-2-oxobutanamide (80 mg, 0.38 mmol), in accordance with General Procedure A, the title compound was obtained (53 mg, 24% yield) as a yellow solid (m.p. 121.2-124.4°C). $^1$H NMR (400 MHz,$CDCl_3$) δ 7.72 (s, 1H), 7.41 (s, 1H), 7.12-7.22 (m, 6H), 7.07-7.13 (m, 5H), 6.87 (d, $J$ = 9.2 Hz, 2H), 6.63 - 6.69 (m, 3H), 6.25 (dd, $J$ = 8.4, 15.6 Hz, 1H), 4.62 (d, $J$ = 8.4 Hz, 1H), 3.69 (s, 3H), 2.30 (s, 3H), 2.18-2.22 (m, 1H), 2.07-2.11 (m, 1H),1.15 (t, $J$ = 7.2 Hz, 3H). $^{13}$C NMR (400 MHz, $CDCl_3$) δ 167.5, 160.5, 158.4, 140.6, 137.7, 136.4, 134.8, 134.7, 130.3, 130.1, 129.5, 129.1, 128.9, 128.6, 127.9, 126.8, 126.1, 125.4, 125.1, 124.5, 121.1, 119.0, 114.4, 80.3, 55.5, 51.8, 27.3, 21.7, 7.9. HRMS (+ESI-TOF) m/z: $[M+H]^+$ Calcd for $C_{35}H_{33}N_3O_4Cl$ 594.2154 ; Found 594.2140.

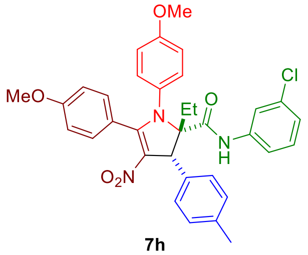

**7h**

**(2RS,3RS)-N-(3-chlorophenyl)-2-ethyl-1,5-bis(4-methoxyphenyl)-4-nitro-3-(p-tolyl)-2,3-dihydro-1H-pyrrole-2-carboxamide (7h)**

Using N-(3-chlorophenyl)-2-oxobutanamide (80 mg, 0.38 mmol), in accordance with General Procedure A, the title compound was obtained (61 mg, 27% yield) as a yellow solid (m.p. 105.5-108.5°C). $^1$H NMR (400 MHz, $CD_3OD$) δ 7.91 (s, 1H), 7.30 (s, 2H), 7.25 (d, $J$ = 8.4 Hz, 2H), 7.07-7.13 (m, 6H), 6.79 (d, $J$ = 8.4 Hz, 4H), 6.68 (d, $J$ = 8.4 Hz, 2H), 4.96 (s, 1H), 3.71 (s, 3H), 3.68 (s, 3H), 2.48-2.53 (m, 1H), 2.31 (s, 3H), 2.17-2.25 (m, 1H), 1.27-1.31 (m, 3H). $^{13}$C NMR (400 MHz, $CD_3OD$) δ 169.0, 165.5, 160.7, 160.2, 141.4, 136.8, 131.6, 131.4, 131.3, 130.6, 130.4, 129.7, 129.2, 128.3, 125.2, 125.1, 114.8, 83.7, 55.83, 55.81, 54.9, 28.8, 21.5, 8.5. HRMS (-ESI-TOF) m/z: $[M-H]^-$ Calcd for $C_{34}H_{31}N_3O_5Cl$ 596.1958; Found 596.1962.

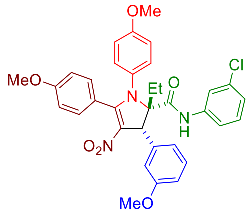


**7i**

**(2RS,3RS)-N-(3-chlorophenyl)-2-ethyl-3-(3-methoxyphenyl)-1,5-bis(4-methoxyphenyl)-4-nitro-2,3-dihydro-1H-pyrrole-2-carboxamide (7i)**

Using N-(3-chlorophenyl)-2-oxobutanamide (80 mg, 0.38 mmol), in accordance with General Procedure A, the title compound was obtained (62 mg, 27% yield) as a yellow solid (m.p. 67.0-69.6°C). $^{1}$H NMR (400 MHz, $d_6$-DMSO) δ 9.06 (s, 1H), 7.27-7.28 (m, 3H), 7.13 - 7.19 (m, 3H), 7.05 (d, $J$ = 7.6 Hz, 2H), 6.88 (t, $J$ = 8.4 Hz, 3H), 6.76 (dd, $J$ = 8.4, 22.8 Hz, 5H), 4.84 (s, 1H), 3.71 (s, 3H), 3.66 (s, 3H), 3.64 (s, 3H), 2.54 - 2.59 (m, 1H), 2.06 - 2.11 (m, 1H), 1.22 (t, $J$ = 6.4 Hz, 3H). $^{13}$C NMR (100 MHz, $d_6$-DMSO) δ 167.1, 162.2, 159.0, 158.5, 148.9, 139.0, 132.7, 131.7, 130.8, 130.5, 130.1, 129.7, 129.6, 124.7, 124.3, 122.6, 121.3, 115.3, 114.1, 113.7, 82.2, 60.2, 55.7, 55.6, 55.5, 53.4, 21.2, 14.6, 8.4. HRMS (+ESI-TOF) m/z: $[M+H]^+$ Calcd for $C_{34}H_{33}N_3O_6Cl$ 614.2052; Found 614.2066.

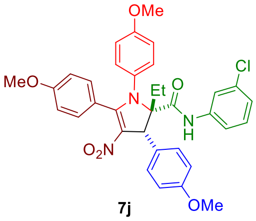


**7j**

**(2RS,3RS)-N-(3-chlorophenyl)-2-ethyl-1,3,5-tris(4-methoxyphenyl)-4-nitro-2,3-dihydro-1H-pyrrole-2-carboxamide (7j)**

Using N-(3-chlorophenyl)-2-oxobutanamide (80 mg, 0.38 mmol), in accordance with General Procedure A, the title compound was obtained (56 mg, 24% yield) as a yellow solid (m.p. 84.2-86.9°C). $^{1}$H NMR (400 MHz, $CD_3OD$) δ 7.39 (d, $J$ = 8.0 Hz, 2H), 7.27 (d, $J$ = 8.4 Hz, 2H), 7.08 - 7.14 (m, 3H), 7.02 - 7.05 (m, 1H), 6.79 - 6.87 (m, 6H), 6.72 (d, $J$ = 8.8 Hz, 2H), 4.97 (s, 1H), 3.77 (s, 3H), 3.72 (s, 3H), 3.68 (s, 3H), 2.48 - 2.57 (m, 1H), 2.15 - 2.28 (m, 1H), 1.27 - 1.31 (m, 3H). $^{13}$C NMR (100 MHz, $CDCl_3$) δ 167.2, 161.1, 160.5, 159.5, 158.3, 137.5, 134.3, 131.6, 131.3, 130.8, 129.7, 129.1, 129.0, 128.8, 125.1, 124.3, 121.1, 121.0, 118.9, 114.43, 114.41, 113.8, 81.2, 55.5, 55.4, 53.9, 29.8, 29.0, 8.2. HRMS (+ESI-TOF) *m/z*: $[M+H]^+$ Calcd for $C_{34}H_{33}N_3O_6Cl$: 614.2052; Found 614.2042.

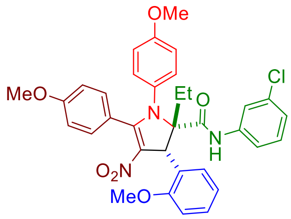


**7k**

**(2RS,3RS)-N-(3-chlorophenyl)-2-ethyl-3-(2-methoxyphenyl)-1,5-bis (4 - methoxyphenyl) - 4 - nitro-2,3 - dihydro - 1H - pyrrole - 2 – carboxamide (7k)**

Using N-(3-chlorophenyl)-2-oxobutanamide (80 mg, 0.38 mmol), in accordance with General Procedure A, the title compound was obtained (41 mg, 18% yield) as a yellow solid (m.p. 67.4-70.3°C). $^{1}$H NMR (400 MHz, $CDCl_3$) δ 8.85 (s, 1H), 7.48 (dd, *J*= 1.6, 7.6 Hz, 1H), 7.39 - 7.44 (m, 1H), 7.30 (t, *J* = 2.0 Hz, 1H), 7.21 (t, *J* = 8.0 Hz, 1H), 7.08 - 7.13 (m, 5H), 6.83 - 6.86 (m, 3H), 6.70 (d, *J* = 7.6 Hz, 2H), 6.60 - 6.63 (m, 2H), 5.24 (s, 1H), 3.694 (s, 3H), 3.691 (s, 3H), 3.58 (s, 3H), 2.35 - 2.40 (m, 1H), 1.94 - 2.00 (m, 1H), 1.05 (t, *J* = 7.6 Hz, 3H). $^{13}$C NMR (100 MHz, $CDCl_3$) δ 167.5, 159.2, 158.1, 157.1, 155.6, 135.9, 132.3, 131.1, 129.9, 129.8, 129.2, 127.3, 127.1, 125.5, 121.9, 121.6, 118.6, 114.0, 113.9, 112.1, 80.6, 58.6, 56.5, 55.5, 55.3, 53.1, 25.5, 18.6, 7.5. HRMS (+ESI-TOF) m/z: $[M+H]^+$ Calcd for $C_{34}H_{33}N_3O_6$ 614.2052; Found 614.2036.

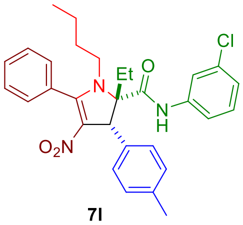


**7l**

**(2RS,3RS)-1-butyl-N-(3-chlorophenyl)-2-ethyl-4-nitro-5-phenyl-3-(p-tolyl)-2,3-dihydro-1H-pyrrole-2-carboxamide (7l)**

A solution of butan-1-amine (34 mg, 0.47 mmol), 4-methylbenzaldehyde (57 mg, 0.47 mmol), N-(3-chlorophenyl)-2-oxobutanamide (100 mg, 0.47 mmol), (E)-(2-nitrovinyl)benzene (70 mg, 0.47 mmol) in anhydrous EtOH (19 ml) was stirred under reflux for 8 h, then extra amine (1.0 equiv) and β-nitroalkene (1.0 equiv) was added to the reaction mixture under reflux and stirring was continued for another 6 h. After cooled down to r.t., ethanol was removed under vacuum, and the residue was diluted with EtOAc and water. After separation, the aqueous phase was extracted two more times with EtOAc. The combined organic phases were successively washed with saturated aqueous $KHSO_4$, saturated aqueous $NaHCO_3$, and brine, dried over $MgSO_4$, concentrated *in vacuo* and purified using gradient elution flash column chromatography on silica gel with petroleum ether/ethyl acetate

(5:1~3:1) to give the the title compound (70 mg, 28% yield) as a yellow solid (m.p. 91.2-94.4°C). $^1$H NMR (400 MHz, $CDCl_3$) δ 7.38 (s, 1H), 7.34 (s, 2H), 7.23-7.22 (m, 2H), 7.17-7.00 (m, 6H), 6.89-6.99 (m, 1H), 6.73-6.67 (m, 1H), 4.74-4.77 (m, 1H), 3.11-3.06 (m, 2H), 2.46 (s, 3H), 2.42-2.35 (m, 1H), 2.21-2.16 (m, 3H), 1.38-1.34 (m, 2H), 1.28-1.23 (m, 4H), 0.99-0.93 (m, 2H), 0.62 (t, $J$ = 7.2 Hz, 3H); $^{13}$C NMR (100 MHz, $CDCl_3$) δ 167.4, 167.2, 164.0, 140.40, 140.35, 137.6, 137.3, 134.2, 134.01, 133.96, 129.8, 129.6, 129.5, 129.4, 128.6, 128.3, 127.8, 127.03, 126.98, 125.0, 124.9, 123.7, 121.7, 121.4, 119.6, 119.3, 81.8, 53.8, 53.5, 46.6, 32.1, 29.5, 29.4, 21.7, 21.1, 20.3, 13.31, 13.27, 8.4. HRMS (+ESI-TOF) m/z: $[M + H]^+$ calcd for $C_{30}H_{33}N_3O_3Cl$ 518.2205; found 518.2209.

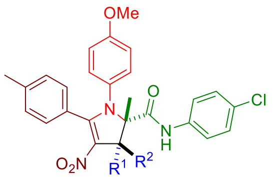


**7m:** R¹ = 4-Me-Ph, R² = H
**7m bis:** R¹ = H, R² = 4-Me-Ph

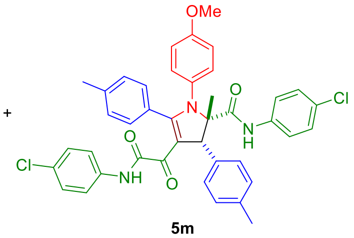

**5m**

**N-(4-chlorophenyl)-1-(4-methoxyphenyl)-2-methyl-4-nitro-3,5-di-p-tolyl-2,3-dihydro-1H-pyrrole-2-carboxamide (7m + 7m bis)**

Using N-(4-chlorophenyl)-2-oxopropanamide (80 mg, 0.40 mmol), in accordance with General Procedure A, the title compound (**7m**) was obtained (81 mg, 35% yield) as a yellow solid (m.p. 125.2-128.3°C). $^1$H NMR (400 MHz, $CDCl_3$) δ 7.28 (s, 1H), 7.16-7.10 (m, 7H), 7.04 (d, $J$ = 7.6 Hz, 2H), 6.91 (d, $J$ = 8.4 Hz, 2H), 6.80 (d, $J$ = 8.4 Hz, 2H), 6.64 (d, $J$ = 8.8 Hz, 2H), 4.86 (s, 1H), 3.69 (s, 3H), 2.32 (s, 3H), 2.21 (s, 3H), 1.77 (s, 3H); $^{13}$C NMR (100 MHz, $CDCl_3$) δ 167.1, 160.4, 158.7, 140.5, 138.0, 135.0, 133.4, 130.6, 130.1, 130.0, 129.7, 129.4, 129.1, 128.7, 127.6, 126.2, 125.0, 122.0, 114.4, 78.2, 59.0, 55.5, 24.4, 21.7, 21.1. HRMS (+ESI-TOF) m/z: $[M + H]^+$ calcd for $C_{33}H_{31}N_3O_4Cl$ 568.1998; found 568.2005.

The diastereoisomeric product (**7m bis**) and the three-component product (**5m**, 27mg, 19% yield) were also separated from the column. **7m bis**: 26 mg, 11% yield, a yellow solid (m.p. 111.2-115.2°C). $^1$H NMR (400 MHz, $CDCl_3$) δ 8.32 (s, 1H), 7.57 (d, 2H, $J$ = 8.4 Hz), 7.37 (t, 4H, $J$ = 8.8 Hz), 7.28 (s, 1H), 7.20 (d, 2H, $J$ = 8.0 Hz), 7.14 (d, 2H, $J$ = 7.6 Hz), 6.88 (d, 2H, $J$ = 8.8 Hz), 6.66 (d, 2H, $J$ = 8.8 Hz), 4.91 (s, 1H), 3.69 (s, 3H), 2.35 (s, 3H), 2.34 (s, 3H), 0.90 (s, 3H); $^{13}$C NMR (100 MHz, $CDCl_3$) δ 171.7, 158.8, 158.7, 141.2, 138.0, 135.9, 133.8, 130.6, 130.4, 130.1, 129.8, 129.4, 129.3, 129.2, 126.2, 126.0, 121.6, 114.6, 75.6, 55.6, 55.5, 21.8, 21.3, 19.5. HRMS (-ESI-TOF) m/z: $[M - H]^-$ calcd for $C_{33}H_{29}N_3O_4Cl$ 566.1852; found 566.1832.

Please refer to our previous publication[1] for the spectra of compound **5m**.

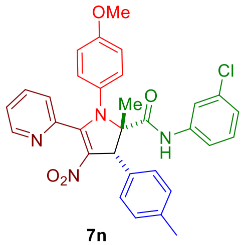


**7n**

**(2RS,3RS)-N-(3-chlorophenyl)-1-(4-methoxyphenyl)-2-methyl-4-nitro-5-(pyridin-2-yl)-3-(p-tolyl)-2,3-dihydro-1H-pyrrole-2-carboxamide (7n)**

Using N-(3-chlorophenyl)-2-oxopropanamide (80 mg, 0.40 mmol), in accordance with General Procedure A, the title compound was obtained (56 mg, 25% yield) as a yellow solid (m.p. 103.0-106.4°C). $^{1}$H NMR (400 MHz, $CDCl_3$) δ 8.42 (d, $J$ = 4.8 Hz, 1H), 8.03 (s, 1H), 7.56-7.60 (m,1H), 7.32 (t, $J$ = 6.4 Hz, 3H), 7.25 (d, $J$ = 5.2 Hz, 1H), 7.09-7.14 (m, 3H), 6.97-7.07 (m, 3H), 6.93 (d, $J$ = 8.8 Hz, 2H), 6.65 (d, $J$ = 9.2 Hz, 2H), 5.03 (s, 1H), 3.69 (s, 3H), 2.31 (s, 3H), 1.83 (s, 3H). $^{13}$C NMR (100 MHz, $CDCl_3$) δ 167.2, 160.7, 158.8, 156.9, 149.0, 140.5, 137.9, 137.3, 134.5, 130.5, 130.2, 129.9, 129.6, 129.0, 126.2, 124.9, 124.6, 124.3, 122.9, 120.1, 118.0, 114.4, 60.7, 55.5, 29.8, 25.1, 21.7. HRMS (+ESI-TOF) m/z: $[M+H]^+$ Calcd for $C_{35}H_{33}N_3O_4Cl$ 594.2154 ; Found 594.2140.

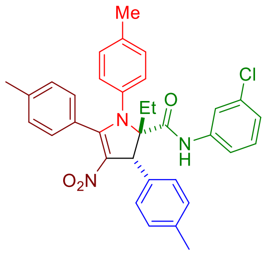


**7o**

**(2RS,3RS)-N-(3-chlorophenyl)-2-ethyl-4-nitro-1,3,5-tri-p-tolyl-2,3-dihydro-1H-pyrrole-2-carboxamide (7o)**

Using N-(3-chlorophenyl)-2-oxobutanamide (80 mg, 0.38 mmol), in accordance with General Procedure A, the title compound was obtained (66 mg, 31% yield) as a yellow solid (m.p. 114.8-117.0°C). $^{1}$H NMR (400 MHz, $CDCl_3$) δ 7.29 (d, $J$ = 10.4 Hz, 2H), 7.12 - 7.18 (m, 4H), 7.04 - 7.07 (m, 3H), 6.99-7.01 (m, 1H), 6.91 - 6.94 (m, 3H), 6.82 (d, $J$ = 8.0 Hz, 2H), 6.69 (s, 1H), 6.62 (d, $J$ = 8.0 Hz, 1H), 5.03 (s, 1H), 2.37 - 2.41 (m, 1H), 2.34 (s, 3H), 2.24 -2.31 (m, 1H), 2.23 (s, 3H), 2.21 (s, 3H), 1.24 (t, $J$ = 7.2 Hz, 3H); $^{13}$C NMR (100 MHz, $CDCl_3$) δ 167.1, 160.5, 140.6, 137.9, 137.4, 137.2, 135.4, 134.2, 133.8, 129.8, 129.61, 129.56, 129.5, 129.1, 127.9, 127.3, 126.2, 125.1, 124.8, 121.3, 119.1, 81.4, 54.4, 29.1, 21.7, 21.1, 21.0, 8.1. HRMS (-ESI-TOF) m/z: $[M - H]^-$ calcd for $C_{34}H_{31}N_3O_3Cl$ 564.2059; found 564.2065.

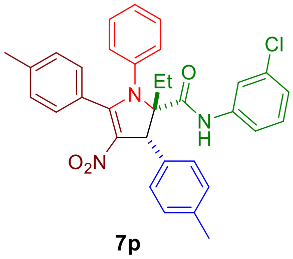


**7p**

**(2RS,3RS)-N-(3-chlorophenyl)-2-ethyl-4-nitro-1-phenyl-3,5-di-p-tolyl-2,3-dihydro-1H-pyrrole-2-carboxamide (7p)**

Using N-(3-chlorophenyl)-2-oxobutanamide (80 mg, 0.38 mmol), in accordance with General Procedure A, the title compound was obtained (44 mg, 21% yield) as a yellow solid (m.p. 68.2-71.3°C). $^1$H NMR (400 MHz, $CDCl_3$) δ 7.30 (d, $J$ = 8.0 Hz, 2H), 7.09 - 7.18 (m, 7H), 7.03 - 7.06 (m, 3H), 6.94 - 7.00 (m，4H), 6.68 (s, 1H), 6.61 (d, $J$ = 8.0 Hz, 2H), 5.03 (s, 1H), 2.33 - 2.43 (m, 1H), 2.29 (s, 3H), 2.21 - 2.28 (m, 4H), 1.21 - 1.29 (m, 3H). $^{13}$C NMR (100 MHz, $CDCl_3$) δ 167.1, 159.8, 140.8, 138.2, 138.1, 137.3, 134.3, 133.7, 129.7, 129.6, 129.3, 129.1, 127.8, 127.3, 127.1, 126.1, 125.2, 121.2, 119.0, 81.3, 54.5, 29.3, 21.7, 21.1, 8.1. HRMS (+ESI-TOF) m/z: $[M + Na]^+$ calcd for $C_{33}H_{30}N_3O_3Cl\,Na$ 574.1868; found 574.1852.

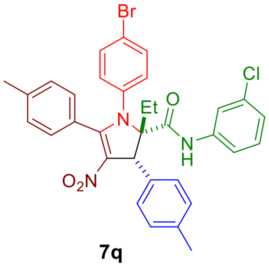


**7q**

**(2RS,3RS)-1-(4-bromophenyl)-N-(3-chlorophenyl)-2-ethyl-4-nitro-3,5-di-p-tolyl-2,3-dihydro-1H-pyrrole-2-carboxamide (7q)**

Using N-(3-chlorophenyl)-2-oxobutanamide (80 mg, 0.38 mmol), in accordance with General Procedure A, the title compound was obtained (21 mg, 9% yield) as a yellow solid (m.p. 104.7-116.8°C). $^1$H NMR (400 MHz, $CDCl_3$) δ 7.21 (d, $J$ = 8.4 Hz, 2H), 7.18 (d, $J$ = 10.0 Hz, 2H), 7.09 (d, $J$ = 9.6 Hz, 4H), 7.00 -7.04 (m, 3H), 6.95 (d, $J$ = 8.4 Hz, 1H), 6.69 -6.94 (m, 3H), 6.59-6.65 (m, 2H), 5.00 (s, 1H), 2.22 - 2.29 (m, 4H), 2.12 -2.18 (m, 4H), 1.14-1.18 (m, 3H). $^{13}$C NMR (100 MHz, $CDCl_3$) δ 167.0, 159.6, 141.0, 138.3, 137.33, 137.28, 134.3, 133.4, 132.2, 129.8, 129.7, 129.6, 129.3, 128.9, 125.8, 125.5, 125.3, 121.3, 120.7, 119.1, 81.2, 54.4, 28.4, 21.8, 21.1, 8.2. HRMS (+ESI-TOF) m/z: $[M + Na]^+$ calcd for $C_{33}H_{29}N_3O_3ClBrNa$ 652.0973; found 652.1005.

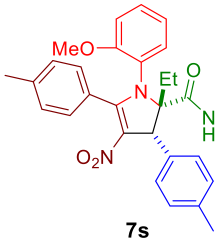


**7s**

**(2RS,3RS)-N-(3-chlorophenyl)-2-ethyl-1-(2-methoxyphenyl)-4-nitro-3,5-di-p-tolyl-2,3-dihydro-1H-pyrrole-2-carboxamide (7s)**

Using N-(3-chlorophenyl)-2-oxobutanamide (80 mg, 0.38 mmol), in accordance with General Procedure A, the title compound was obtained (72 mg, 33% yield) as a yellow solid (m.p. 177.5-180.5°C). $^1$H NMR (400 MHz, $CDCl_3$) δ 7.85 (s, 1H), 7.09-7.22 (m, 4H), 7.09 (t, $J$ = 7.6 Hz, 2H), 7.01 (d, $J$ = 8.0 Hz, 3H), 6.89 (q, $J$ = 8.4 Hz, 3H), 6.82 (d, $J$ = 8.0 Hz, 1H), 6.52 (d, $J$ = 8.4 Hz, 1H), 6.47 (s, 1H), 6.37 (d, 8.0 Hz, 1H), 4.68 (s, 1H)， 3.54 (s, 3H), 2.59 - 2.65 (m, 1H), 2.38 - 2.44 (m, 1H), 2.23 (s, 3H), 2.11 (s, 3H), 1.38 (t, $J$ = 7.6 Hz, 3H). $^{13}$C NMR (100 MHz, $CDCl_3$) δ 166.8, 161.0, 153.4, 140.7, 137.9, 137.7, 134.8, 134.0, 130.1, 129.4, 129.1, 128.1, 127.1, 125.8, 124.6, 124.2, 122.3, 120.8, 118.4, 111.9, 81.8, 55.7, 55.3, 32.8, 21.7, 21.0, 7.9. HRMS (+ESI-TOF) m/z: $[M + Na]^+$ calcd for $C_{34}H_{32}N_3O_4ClNa$ 604.1971; found 604.1963.

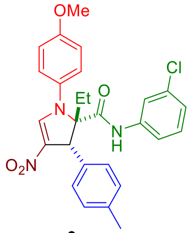


**9**

**(2RS,3RS)-N-(3-chlorophenyl)-2-ethyl-1-(4-methoxyphenyl)-4-nitro-3-(p-tolyl)-2,3-dihydro-1H-pyrrole-2-carboxamide (9)**

A solution of 4-methoxyaniline (58 mg, 0.47 mmol), 4-methylbenzaldehyde (57 mg, 0.47mmol), N-(3-chlorophenyl)-2-oxobutanamide (100 mg, 0.47 mmol), 2-nitroethyl pivalate (83 mg, 0.47 mmol) and acetic acid (1.6 ml) in anhydrous EtOH (18.9 ml, 0.025 mol/L) was stirred under reflux for 8 h. After cooled down to r.t., ethanol was removed under vacuum, and the residue was diluted with EtOAc and water. After separation, the aqueous phase was extracted two more times with EtOAc. The combined organic phases were successively washed with saturated aqueous $KHSO_4$, saturated aqueous $NaHCO_3$, and brine, dried over $MgSO_4$, concentrated in vacuo and purified using gradient elution flash column chromatography on silica gel with petroleum ether/ethyl acetate (5:1~3:1) to give the desired product (84 mg, 36% yield) as a yellow solid (m.p. 160.6-164.1°C). $^1$H NMR (400

MHz, $CDCl_3$) δ 9.07 (s, 1H), 7.75-7.74 (m, 1H), 7.48 (dd, *J* = 0.8, 8.0 Hz, 1H), 7.23 (d, *J* = 8.0 Hz, 1H), 7.16-7.10 (m, 3H), 6.93 (d, *J* = 7.6 Hz, 2H), 6.78 (d, *J* = 8.8 Hz, 2H), 6.60 (d, *J* = 9.2 Hz, 2H), 3.67 (s, 3H), 3.47 (d, *J* = 16.4 Hz, 1H), 2.23 (s, 3H), 2.05-1.98 (m, 1H), 1.87-1.79 (m, 1H), 1.06 (t, *J* = 7.2 Hz, 3H); $^{13}C$ NMR (100 MHz, $CDCl_3$) δ 168.9, 161.3, 159.0, 140.3, 139.2, 134.5, 129.9, 129.8, 129.6, 129.3, 128.7, 125.8, 124.8, 121.4, 121.1, 119.1, 114.3, 75.7, 55.4, 36.9, 27.9, 21.7, 7.5. HRMS (-ESI-TOF) m/z: $[M - H]^-$ calcd for $C_{27}H_{25}N_3O_4Cl$ 490.1539; found 490.1552.

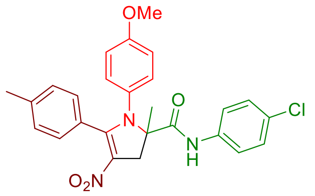


**11a**

**(RS)-N-(4-chlorophenyl)-1-(4-methoxyphenyl)-2-methyl-4-nitro-5-(p-tolyl)-2,3-dihydro-1H-pyrrole-2-carboxamide (11a)**

Using (E)-2-nitro-3-(p-tolyl)prop-2-en-1-ol (98 mg, 0.50 mmol), N-(4-chlorophenyl)-2-oxopropanamide (100 mg, 0.50 mmol), 4-methoxyaniline (61 mg, 0.50 mmol) and HOAc (1.6 ml) in EtOH (20 ml), in accordance with General Procedure B, the title compound was obtained (31 mg, 13% yield) as a yellow solid (m.p. 220.9-223.6°C). $^1H$ NMR (400 MHz, $CDCl_3$) δ 8.90 (s, 1H), 7.63 (d, *J* = 8.8 Hz, 2H), 7.32 (d, *J* = 8.4 Hz, 2H), 7.16 (d, *J* = 7.6 Hz, 2H), 6.92 (d, *J* = 8.0 Hz, 2H), 6.76 (d, *J* = 8.8 Hz, 2H), 6.60 (d, *J* = 8.4 Hz, 2H), 3.67 (s, 3H), 3.63 (d, *J* = 16.0 Hz, 1H), 3.32 (d, *J* = 16.0 Hz, 1H), 2.23 (s, 3H), 1.46 (s, 3H); $^{13}C$ NMR (100 MHz, $CDCl_3$) δ 169.6, 160.4, 159.0, 140.6, 136.6, 130.0, 129.9, 129.8, 129.7, 129.1, 128.7, 125.7, 121.9, 121.6, 114.4, 72.3, 55.5, 41.5, 23.5, 21.7. HRMS (-ESI-TOF) m/z: $[M - H]^-$ calcd for $C_{26}H_{23}N_3O_4Cl$ 476.1383; found 476.1384.

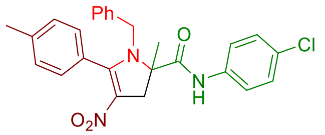


**11b**

**(RS)-N-(4-chlorophenyl)-2-methyl-4-nitro-1-phenethyl-5-(p-tolyl)-2,3-dihydro-1H-pyrrole-2-carboxamide (11b)**

Using (E)-2-nitro-3-(p-tolyl)prop-2-en-1-ol (98 mg, 0.50 mmol), N-(4-chlorophenyl)-2-oxopropanamide (100 mg, 0.50 mmol), benzyl amine (62 mg, 0.50 mmol) and HOAc (1.6 ml) in EtOH (20 ml), in accordance with General Procedure B, the title compound was obtained (65 mg, 28% yield) as a yellow solid (m.p. 85.1-88.9°C). $^1H$ NMR (400 MHz, $CDCl_3$) δ 8.36 (s, 1H), 7.33-7.30 (m, 3H), 7.21-7.17 (m, 5H), 7.14-7.08 (m, 3H), 7.07-7.02 (m, 2H), 4.42-4.29 (m, 2H), 3.65 (d, *J*

= 15.6 Hz, 1H), 3.27 (d, *J* = 16.0 Hz, 1H), 2.33 (s, 3H), 1.65 (s, 3H); $^{13}$C NMR (100 MHz, $CDCl_3$) δ 169.2, 162.3, 140.6, 136.2, 135.7, 129.7, 129.6, 128.8, 128.7, 128.1, 128.0, 127.7, 126.3, 121.7, 121.1, 71.6, 48.7, 41.3, 23.5, 21.6. HRMS (+ESI-TOF) m/z: $[M + H]^+$ calcd for $C_{26}H_{25}N_3O_3Cl$ 462.1579; found 462.1575.

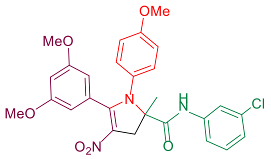


**11c**

**(RS)-N-(3-chlorophenyl)-5-(3,5-dimethoxyphenyl)-1-(4-methoxyphenyl)-2-methyl-4-nitro-2,3-dihydro-1H-pyrrole-2-carboxamide (11c)**

Using (E)-3-(3,5 - dimethoxyphenyl)-2-nitroprop-2-en-1-ol (363 mg, 1.5 mmol), N-(3-chlorophenyl)-2-oxopropanamide (300 mg, 1.5 mmol), 4-methoxyaniline (187 mg, 1.5 mmol) and HOAc (5 ml) in EtOH (60 ml), in accordance with General Procedure B, the title compound was obtained (298 mg, 19% yield) as a yellow solid (m.p. 182.6-186.6°C). $^1$H NMR (400 MHz, $CDCl_3$) δ 8.64 (s, 1H), 7.77 (t, *J* = 2.0 Hz, 1H), 7.58 (d, *J* = 8.8 Hz, 1H), 7.47 (d, *J* = 8.0 Hz, 1H), 7.28 - 7.33 (m, 1H), 7.15 (d, *J* = 7.6 Hz, 1H), 6.84 (d, *J* = 9.2 Hz, 2H), 6.65 (d, *J* = 9.2 Hz, 2H), 6.42 (d, *J* = 1.6 Hz, 2H), 6.31(s, 1H), 3.75 (d, *J* = 16.0 Hz, 1H), 3.70 (s, 3H), 6.65 (s, 6H), 3.39 (d, *J* = 16.0 Hz, 1H), 1.51 (s, 3H). $^{13}$C NMR (100 MHz, $CDCl_3$) δ 169.6, 160.3, 159.4, 159.2, 138.8, 134.9, 130.4, 130.2, 129.8, 129.6, 129.2, 125.1, 121.9, 120.7, 118.6, 114.5, 110.8, 102.5, 72.3, 55.6, 55.5, 41.6, 23.4. HRMS (-ESI-TOF) *m/z*: $[M-H]^-$ Calcd for $C_{27}H_{25}N_3O_6Cl$ 522.1437; Found 522.1439.

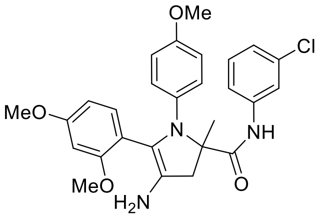


**13a**

**(RS)-4-amino-N-(3-chlorophenyl)-5-(2,4-dimethoxyphenyl)-1-(4-methoxyphenyl)-2-methyl-2,3-dihydro-1H-pyrrole-2-carboxamide (13a)**

To a solution of compound **11c** (490 mg, 0.94 mmol) in $CH_3CN$ (4.68 mL) was added $TiCl_3$ (5.77 ml, 7.48 mmol, 20 wt % in 2 N HCl solution) and the reaction mixture was stirred at room temperature until the total consumption of the starting material (1.5 h). The reaction mixture was then poured into saturated aqueous $NaHCO_3$ solution and extracted with EtOAc thoroughly. The combined organic layer was washed with brine and dried over $Na_2SO_4$. The solvents were removed in vacuo and the

residue was purified by silica gel flash column chromatography to afford both the title compound (**13a**) (64 mg, 14% yield) as a yellow solid (m.p. 71.9-73.4°C), and compound **14a** (30 mg, 6 % yield) as the byproduct. $^{1}$H NMR (400 MHz, $CDCl_3$) δ 10.30 (s, 1H), 7.84 (t, *J* = 2.0 Hz, 1H), 7.49-7.52 (m, 1H), 7.23 (d, *J* = 8.0 Hz, 1H), 7.01-7.04 (m, 3H), 6.85 (d, *J* = 8.8 Hz, 2H), 6.29 (t, *J* = 2.4 Hz, 1H), 6.20 (d, *J* = 2.0 Hz, 2H), 3.80 (s, 3H), 3.64 (s, 6H), 3.28 (s, 2H), 2.45(s, 3H), 1.68-1.75 (m, 1H), 1.25-1.47 (m,1H). $^{13}$C NMR (100 MHz, $CDCl_3$) δ 205.9, 172.5, 161.7, 154.3, 139.3, 138.7, 137.2, 135.1, 130.4, 124.9, 119.9, 118.7, 117.7, 114.9, 104.6, 99.6, 70.1, 65.9, 55.5, 51.0, 17.1. HRMS (+ESI-TOF) m/z: $[M+Na]^+$ Calcd for $C_{27}H_{28}N_3O_4ClNa$ 516.1661; Found 516.1651.

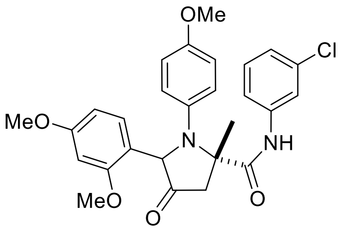


**14a**

**(2RS,5RS)-N-(3-chlorophenyl)-5-(2,4-dimethoxyphenyl)-1-(4-methoxyphenyl)-2-methyl-4-oxopyrrolidine-2-carboxamide (14a)**

To a solution of compound **11c** (80 mg, 0.15 mmol) in $CH_3CN$ (0.38 mL) was added $TiCl_3$ (0.94 ml, 1.2 mmol, 20 wt % in 2 N HCl solution) and the reaction mixture was stirred at room temperature until the total consumption of the starting material (14 h). The reaction mixture was then poured into saturated aqueous $NaHCO_3$ solution and extracted with EtOAc thoroughly. The combined organic layer was washed with brine and dried over $Na_2SO_4$. The solvents were removed in vacuo and the residue was purified by silica gel flash column chromatography to afford the title compound **14a** (23 mg, 30% yield) as a yellow solid (m.p. 130.8-133.0°C). $^{1}$H NMR (400 MHz, $CDCl_3$) δ 9.17 (s, 1H), 7.70 (t, *J* = 2.0 Hz, 1H), 7.39 (d, *J* = 8.0 Hz, 1H), 7.29 (d, *J* = 8.0 Hz, 1H), 7.12 - 7.14 (m, 1H), 6.77 (d, *J* = 9.2 Hz, 2H), 6.63 - 6.68 (m, 4H), 6.44 (t, *J* = 2.0 Hz, 1H), 4.64 (s, 1H), 3.82 (s, 6H), 3.71 (s, 3H), 3.06 (t, *J* = 18.0 Hz, 1H), 2.84 (d, *J* = 17.6 Hz, 1H), 1.50 (s, 3H). $^{13}$C NMR (100 MHz, $CDCl_3$) δ 205.9, 172.5, 161.7, 154.4, 139.4, 138.7, 137.2, 135.1, 130.4, 124.9, 120.0, 118.8, 117.7, 115.0, 104.6, 99.6, 70.1, 65.9, 55.60, 55.55, 51.0, 17.1. HRMS (+ESI-TOF) m/z: $[M+Na]^+$ Calcd for $C_{27}H_{27}N_2O_5ClNa$ 517.1495; Found 517.1501.

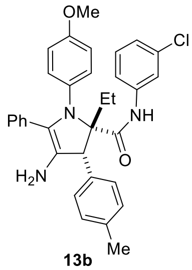

13b

**(2RS,3RS)-4-amino-N-(3-chlorophenyl)-2-ethyl-1-(4-methoxyphenyl)-5-phenyl-3-(p-tolyl)-2,3-dihydro-1H-pyrrole-2-carboxamide (13b)**

To a solution of compound 7**e** (193 mg, 0.34 mmol) in $CH_3CN$ (1.7 mL) was added $TiCl_3$ (2.10 ml, 2.72 mmol, 20 wt % in 2 N HCl solution) and the reaction mixture was stirred at room temperature until the total consumption of the starting materia (2.5 h). The reaction mixture was then poured into saturated aqueous $NaHCO_3$ solution and extracted with EtOAc thoroughly. The combined organic layer was washed with brine and dried over $Na_2SO_4$. The solvents were removed in vacuo and the residue was purified by silica gel flash column chromatography to afford the title compound (68 mg, 37% yield) as a yellow solid (m.p. 66.0-71.5°C). $^1$H NMR (400 MHz,$CD_3OD$) δ 7.89 (s, 2H), 7.77 (s, 1H), 7.54 (d, $J$ = 8.0 Hz, 2H), 7.33-7.37 (m, 2H), 7.25 - 7.31 (m, 7H), 7.11 - 7.13 (m, 1H), 6.71- 6.78 (m, 4H), 5.68 (s, 1H), 3.64 (s, 3H), 2.34 (s, 3H), 2.23 - 2.31 (m, 1H), 1.95 - 2.04 (m, 1H), 0.69 (t, $J$ = 7.2 Hz, 3H). $^{13}$C NMR (100 MHz, $CD_3OD$) δ 176.4, 154.5, 143.9, 140.7, 139.5, 138.5, 138.3, 135.8, 135.0, 131.5, 130.9, 130.8, 129.9, 128.9, 128.3, 125.2, 120.3, 119.0, 118.5, 115.4, 108.3, 81.1, 71.0, 55.9, 24.1, 21.2, 7.0. HRMS (+ESI-TOF) m/z: $[M+Na]^+$ Calcd for $C_{33}H_{32}N_3O_2ClNa$ 560.2075; Found 560.2080.

## 3. NOESY for 7m, 7m-bis and 14a

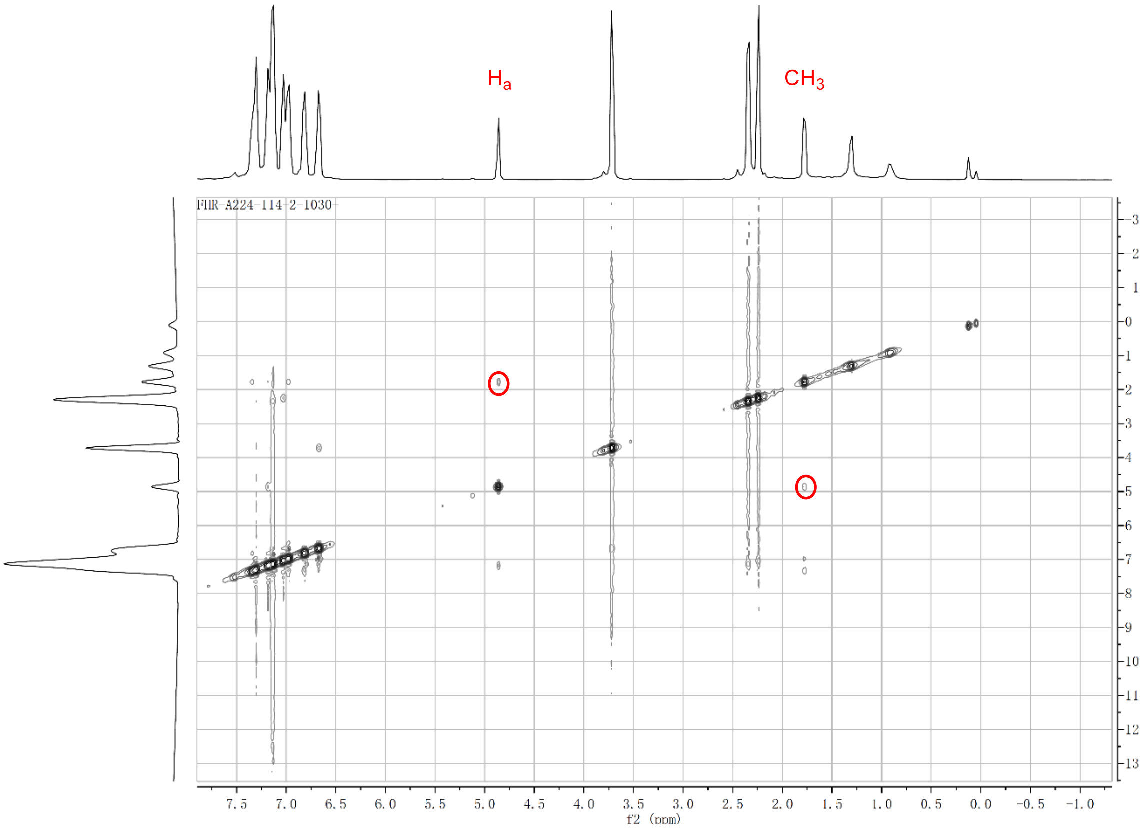


The following correlation was observed:

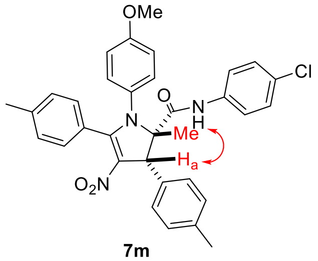


**7m**

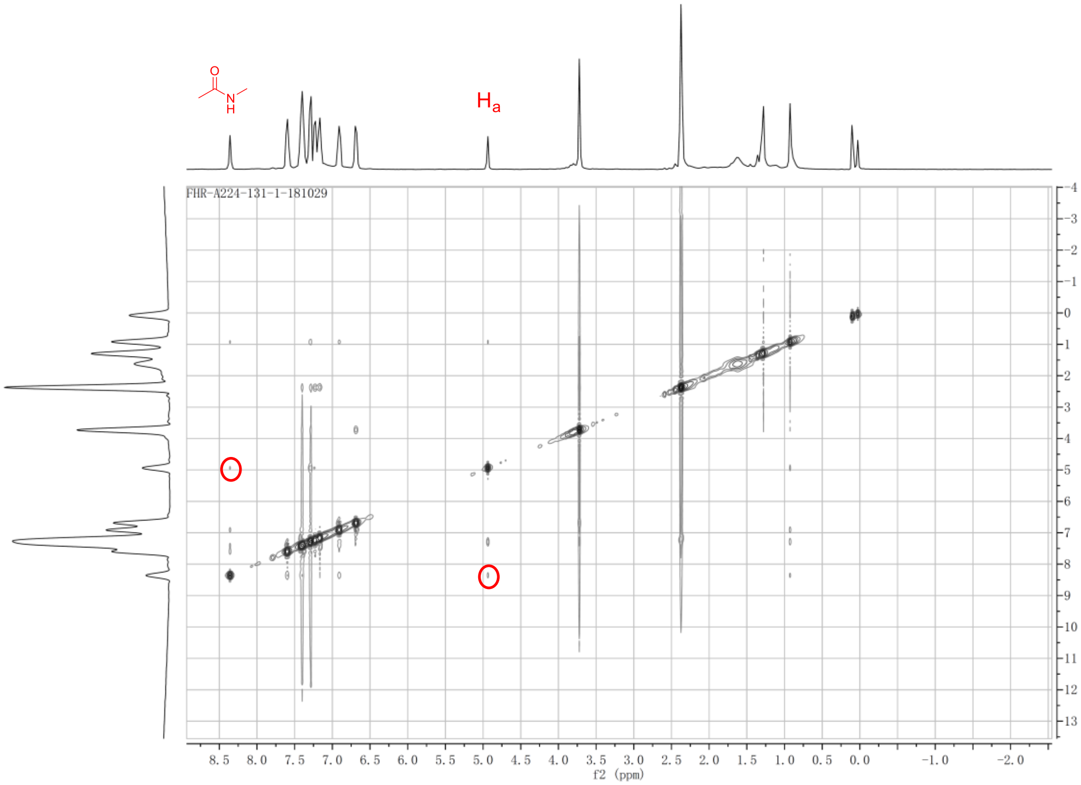


The following correlation was observed:

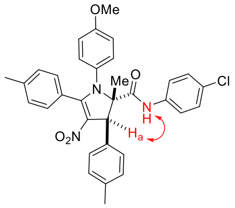


**7m bis**

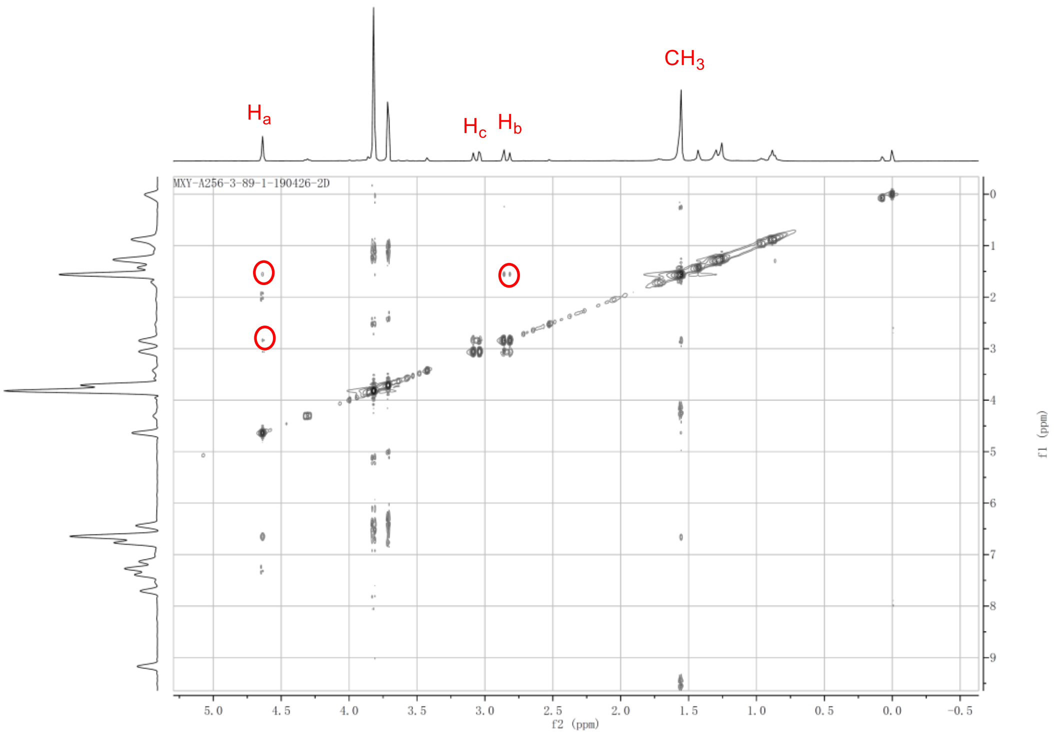


The following correlation was observed:

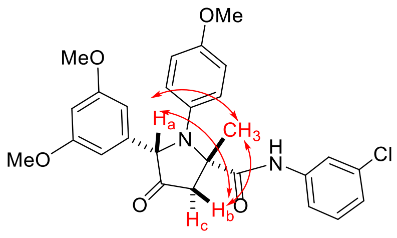


**14a**

# 4. Reference


1. D. Wang, L. Li, H. Feng, H. Sun, F. Almeida-Veloso, M. Charavin, P. Yu and L. Desaubry, *Green Chem.*, 2018, **20**, 2775-2780.


# 5. Spectra Data

Spectra data are shown from the next page.

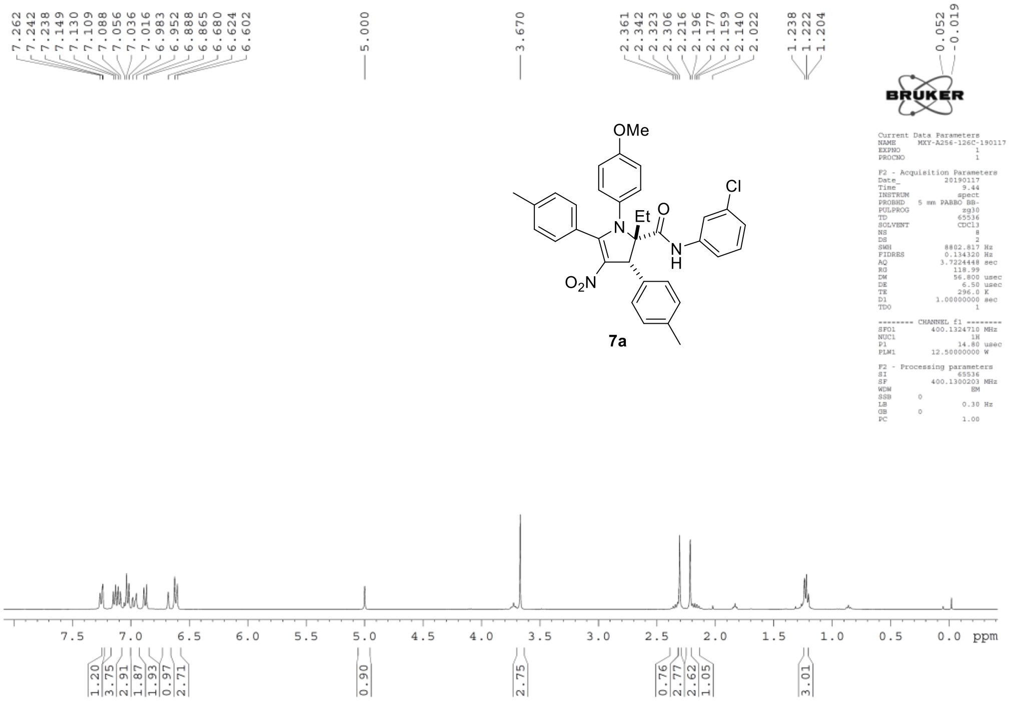
7.262
7.242
7.238
7.149
7.130
7.109
7.088
7.056
7.036
7.016
6.983
6.952
6.888
6.865
6.680
6.624
6.602
5.000
3.670
2.361
2.342
2.323
2.306
2.216
2.196
2.177
2.159
2.140
2.022
1.238
1.222
1.204
0.052
-0.019
OMe
Et
O
Cl
N
N
H
$O_2N$
Current Data Parameters
NAME MXY-A256-126C-190117
EXPNO 1
PROCNO 1
F2 - Acquisition Parameters
Date_ 20190117
Time 9.44
INSTRUM spect
PROBHD 5 mm PABBO BB-
PULPROG zg30
TD 65536
SOLVENT CDCl3
NS 8
DS 2
SWH 8802.817 Hz
FIDRES 0.134320 Hz
AQ 3.7224448 sec
RG 118.99
DW 56.800 usec
DE 6.50 usec
TE 296.0 K
D1 1.00000000 sec
TD0 1
======== CHANNEL f1 ========
SFO1 400.1324710 MHz
NUC1 1H
P1 14.80 usec
PLW1 12.50000000 W
F2 - Processing parameters
SI 65536
SF 400.1300203 MHz
WDW EM
SSB 0
LB 0.30 Hz
GB 0
PC 1.00
7.5 7.0 6.5 6.0 5.5 5.0 4.5 4.0 3.5 3.0 2.5 2.0 1.5 1.0 0.5 0.0 ppm
1.20
3.75
2.91
1.87
1.93
0.97
2.71
0.90
2.75
0.76
2.77
2.62
1.05
3.01

**7a**

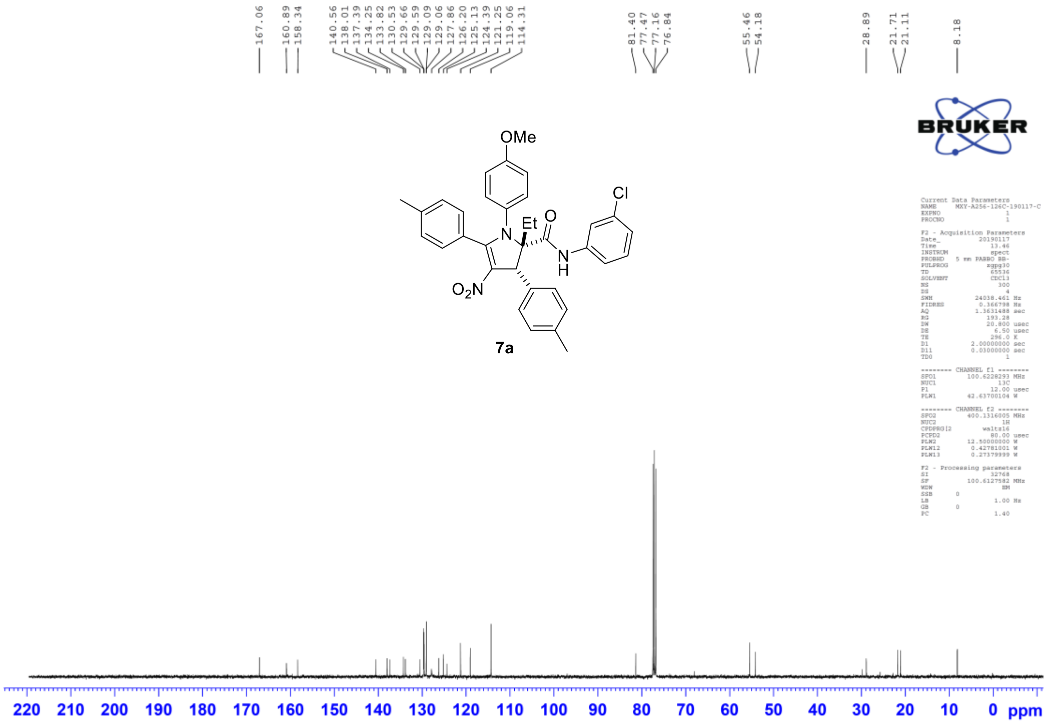
167.06
160.89
158.34
140.56
138.01
137.39
134.25
133.82
130.53
129.66
129.59
129.09
129.06
127.86
126.20
125.13
124.39
121.25
119.06
114.31
81.40
77.47
77.16
76.84
55.46
54.18
28.89
21.71
21.11
8.18
OMe
Et
O
Cl
N
N
H
$O_2N$
Current Data Parameters
NAME MXY-A256-126C-190117-C
EXPNO 1
PROCNO 1
F2 - Acquisition Parameters
Date_ 20190117
Time 13.46
INSTRUM spect
PROBHD 5 mm PABBO BB-
PULPROG zgpg30
TD 65536
SOLVENT CDCl3
NS 300
DS 4
SWH 24038.461 Hz
FIDRES 0.366798 Hz
AQ 1.3631488 sec
RG 193.28
DW 20.800 usec
DE 6.50 usec
TE 296.0 K
D1 2.00000000 sec
D11 0.03000000 sec
TD0 1
======== CHANNEL f1 ========
SFO1 100.6228293 MHz
NUC1 13C
P1 12.00 usec
PLW1 42.63700104 W
======== CHANNEL f2 ========
SFO2 400.1316005 MHz
NUC2 1H
CPDPRG[2 waltz16
PCPD2 80.00 usec
PLW2 12.50000000 W
PLW12 0.42781001 W
PLW13 0.27379999 W
F2 - Processing parameters
SI 32768
SF 100.6127582 MHz
WDW EM
SSB 0
LB 1.00 Hz
GB 0
PC 1.40
220 210 200 190 180 170 160 150 140 130 120 110 100 90 80 70 60 50 40 30 20 10 0 ppm

**7a**

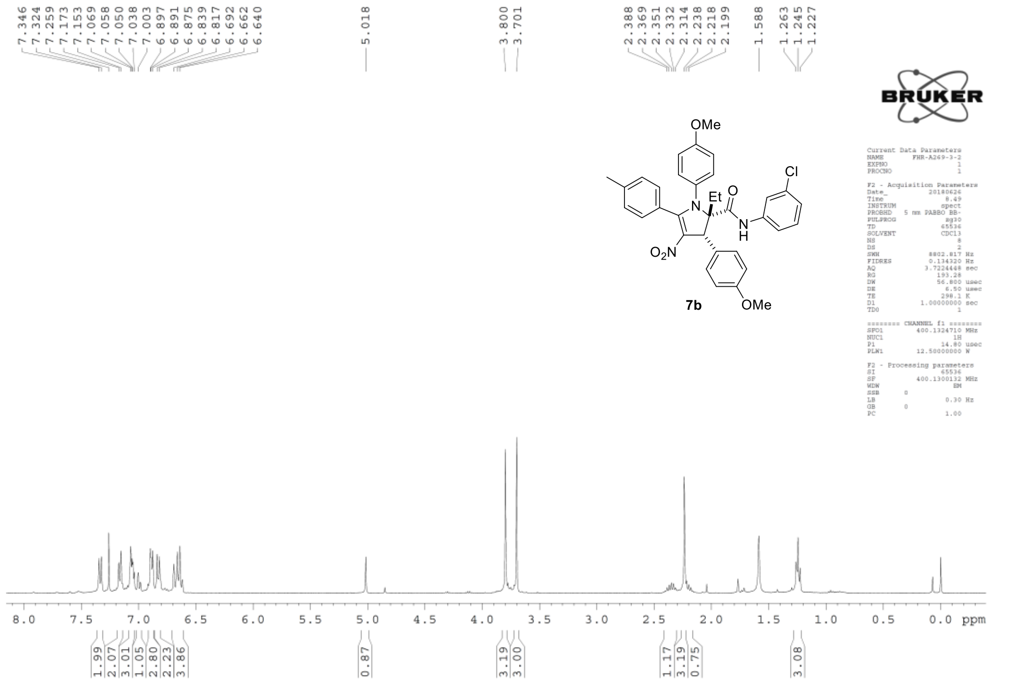
7b
OMe
Cl
Et
O
N
H
$O_2N$
8.0 7.5 7.0 6.5 6.0 5.5 5.0 4.5 4.0 3.5 3.0 2.5 2.0 1.5 1.0 0.5 0.0 ppm

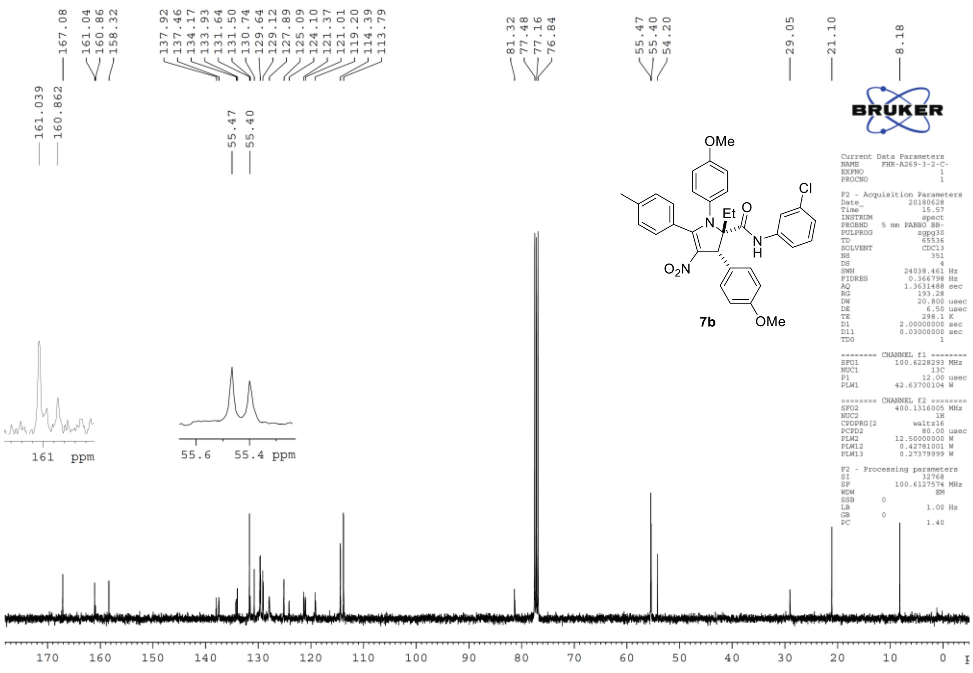
7b
OMe
Cl
Et
O
N
H
$O_2N$
161 ppm
55.6 55.4 ppm
170 160 150 140 130 120 110 100 90 80 70 60 50 40 30 20 10 0

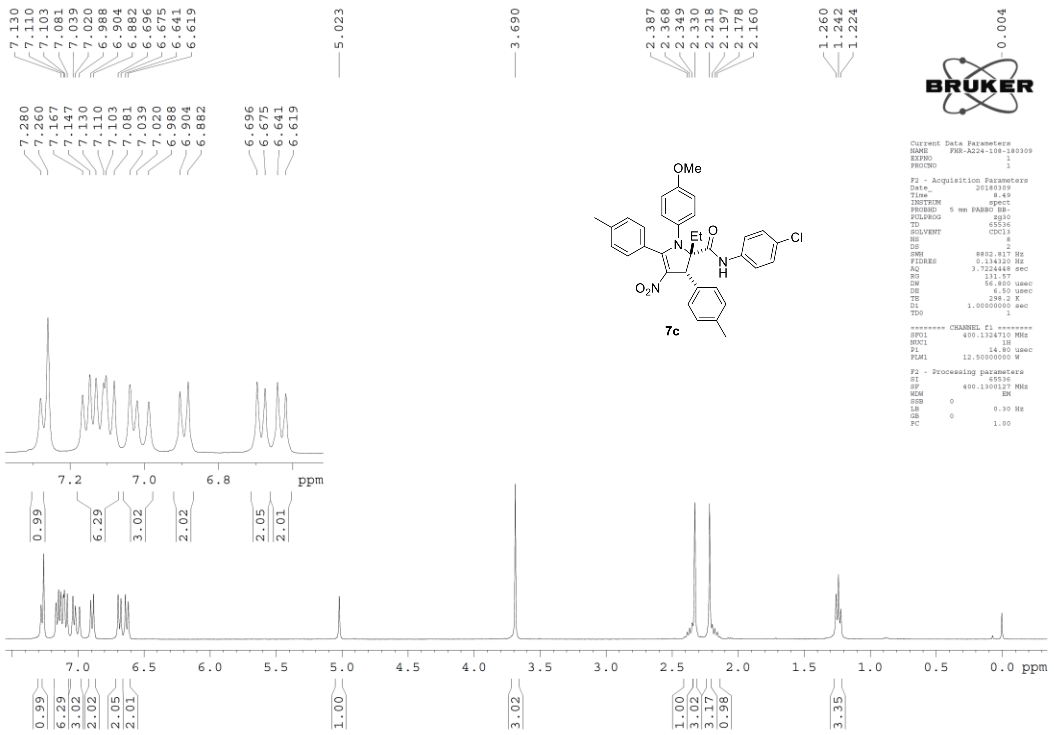

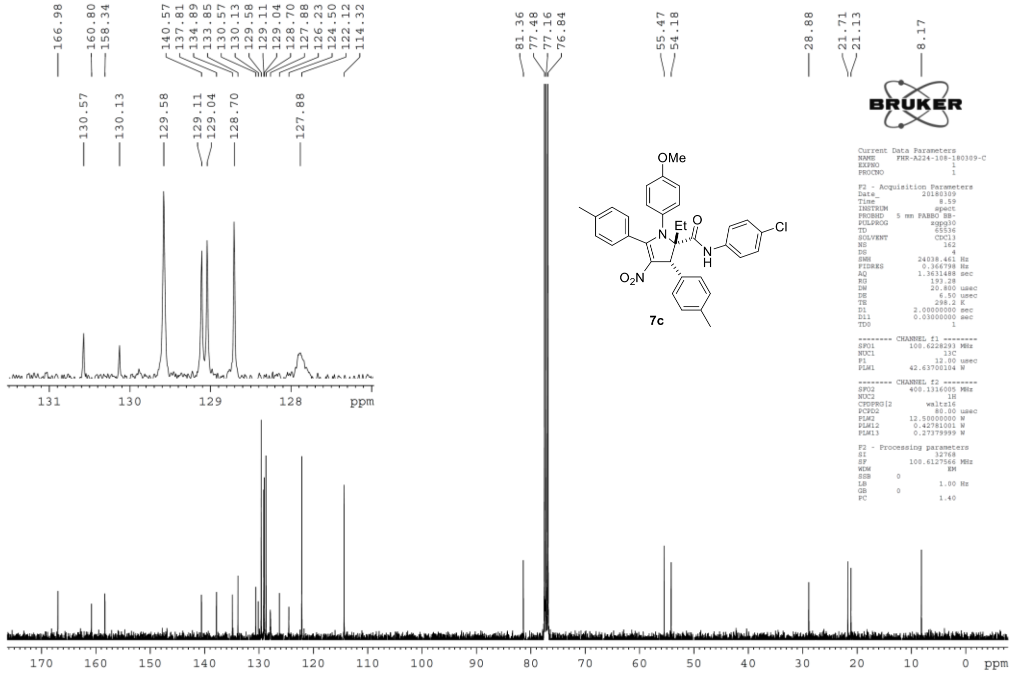

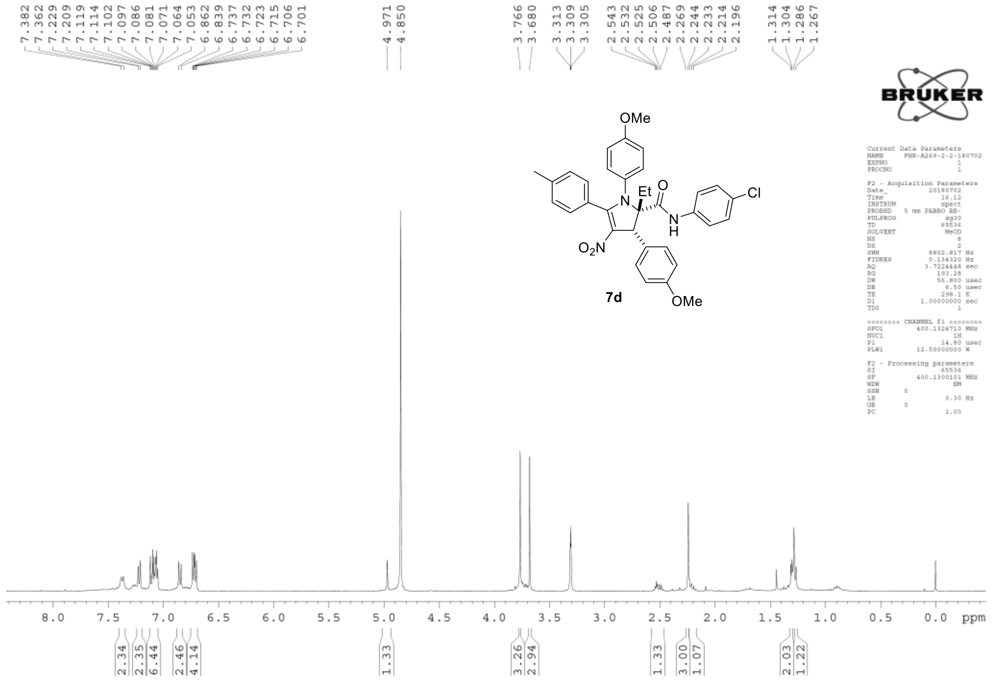
OMe
Et
O
Cl
N
H
$O_2N$
OMe
7d
Current Data Parameters
NAME FHR-A269-2-2-180702
F2 - Acquisition Parameters
ppm

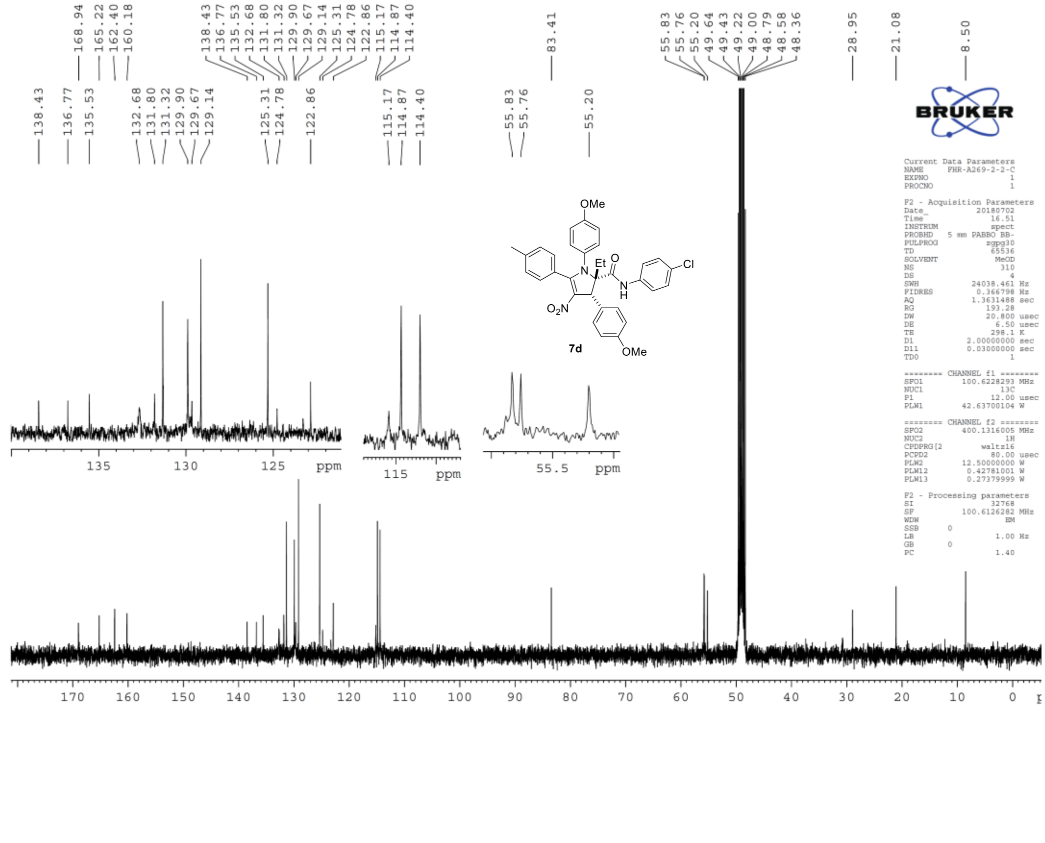
OMe
Et
O
Cl
N
H
$O_2N$
OMe
7d
Current Data Parameters
NAME FHR-A269-2-2-C
F2 - Acquisition Parameters
F2 - Processing parameters
ppm

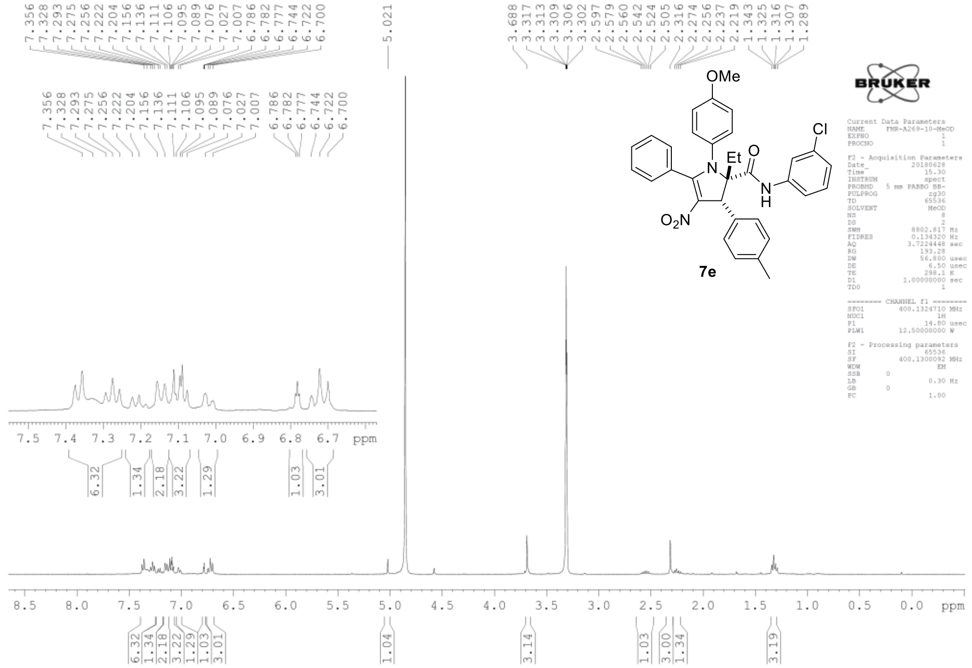

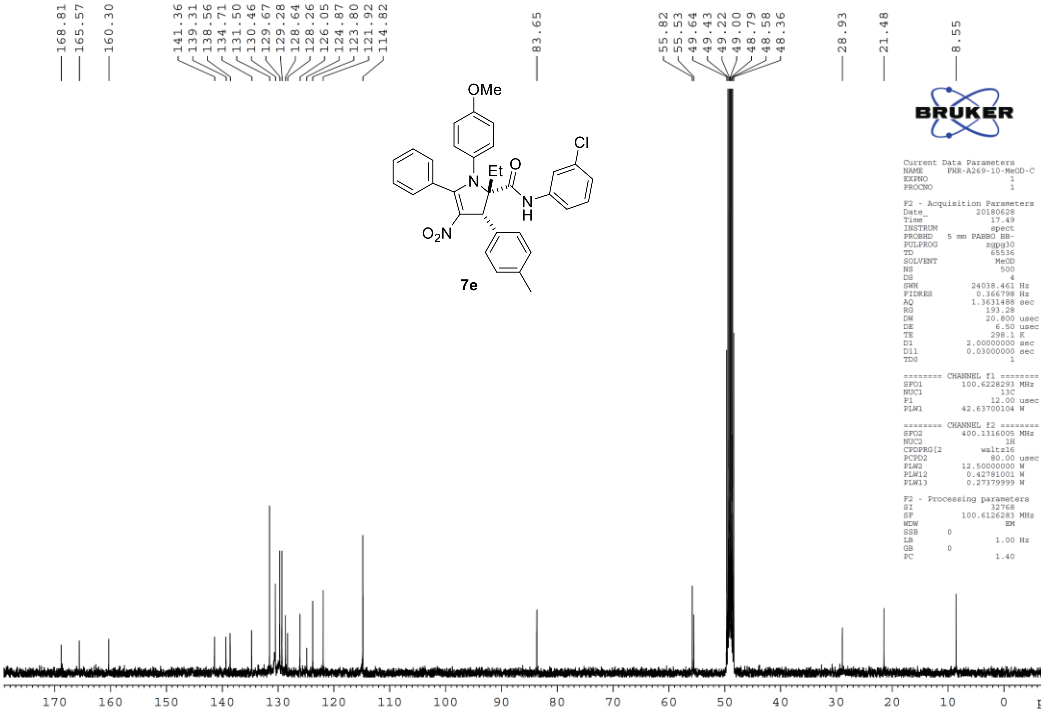

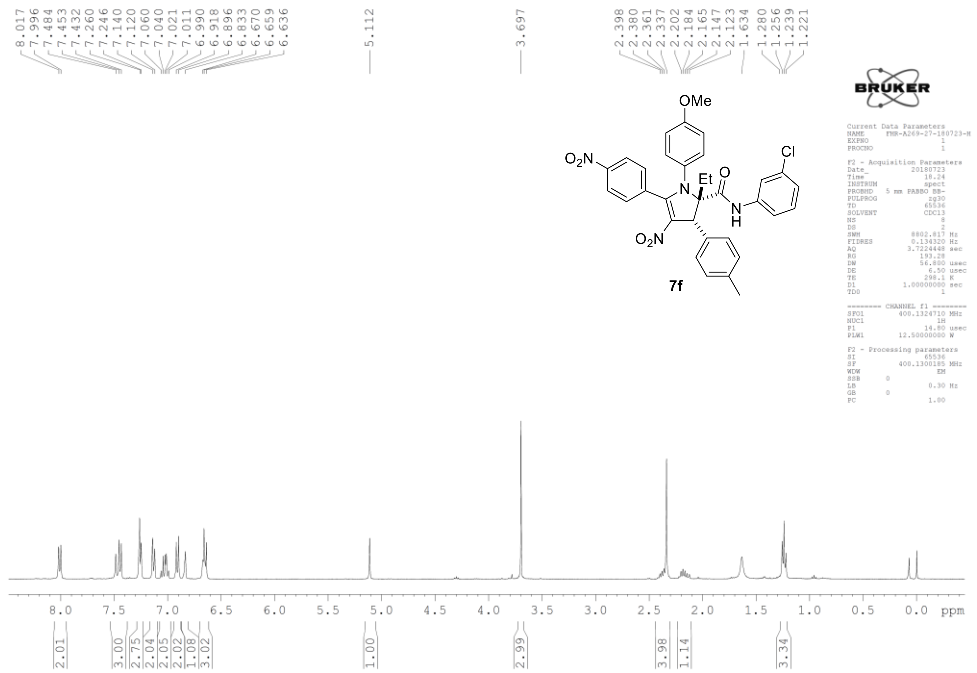
8.017 7.996 7.484 7.453 7.432 7.260 7.246 7.140 7.120 7.060 7.040 7.021 7.011 6.990 6.918 6.896 6.833 6.670 6.659 6.636
5.112
3.697
2.398 2.380 2.361 2.337 2.202 2.184 2.165 2.147 2.123 1.634 1.280 1.256 1.239 1.221
OMe
O2N
Et
O
Cl
N
H
O2N
7f
2.01 3.00 2.75 2.04 2.05 2.02 1.08 3.02 1.00 2.99 3.98 1.14 3.34
ppm

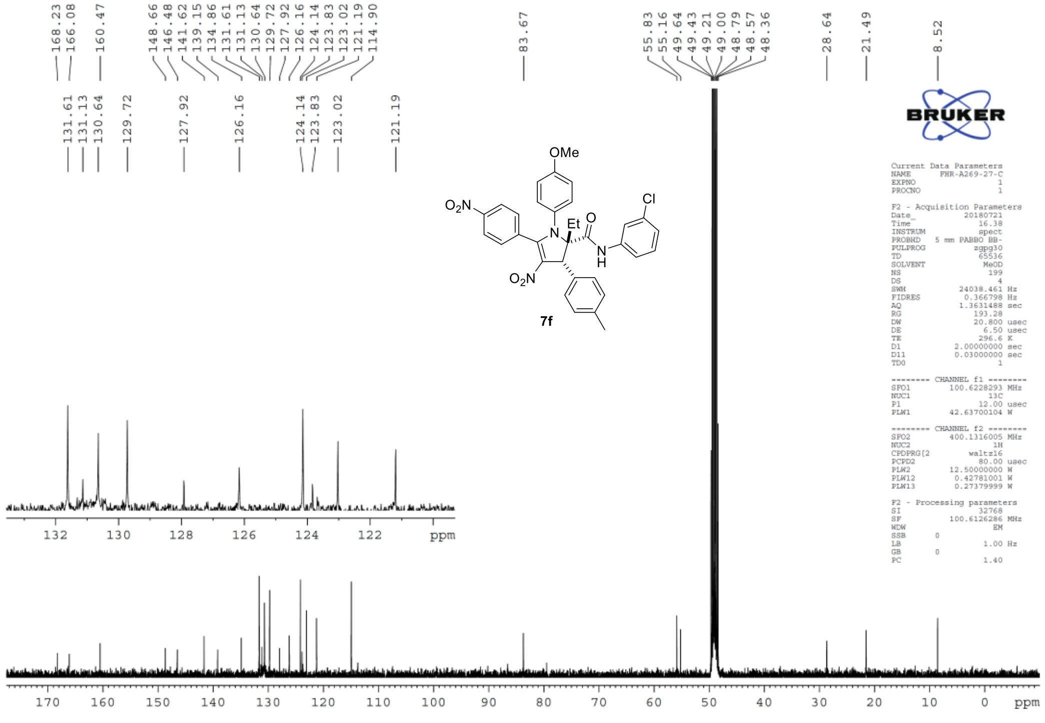
168.23 166.08 160.47 148.66 146.48 141.62 139.15 134.86 131.61 131.13 130.64 129.72 127.92 126.16 124.14 123.83 123.02 121.19 114.90
83.67
55.83 55.16 49.64 49.43 49.21 49.00 48.79 48.57 48.36
28.64
21.49
8.52
131.61 131.13 130.64 129.72 127.92 126.16 124.14 123.83 123.02 121.19
OMe
O2N
Et
O
Cl
N
H
O2N
7f
ppm

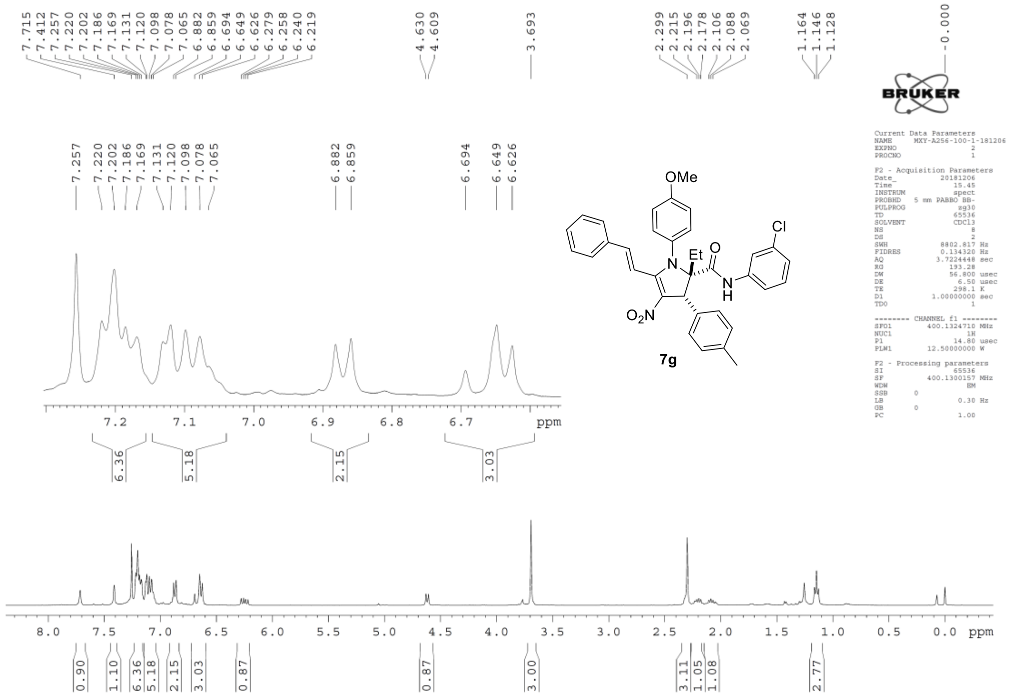
7.715
7.412
7.257
7.220
7.202
7.186
7.169
7.131
7.120
7.098
7.078
7.065
6.882
6.859
6.694
6.649
6.626
6.279
6.258
6.240
6.219
4.630
4.609
3.693
2.299
2.215
2.196
2.178
2.106
2.088
2.069
1.164
1.146
1.128
-0.000
BRUKER
OMe
Cl
N
Et
O
H
$O_2N$
7g
ppm

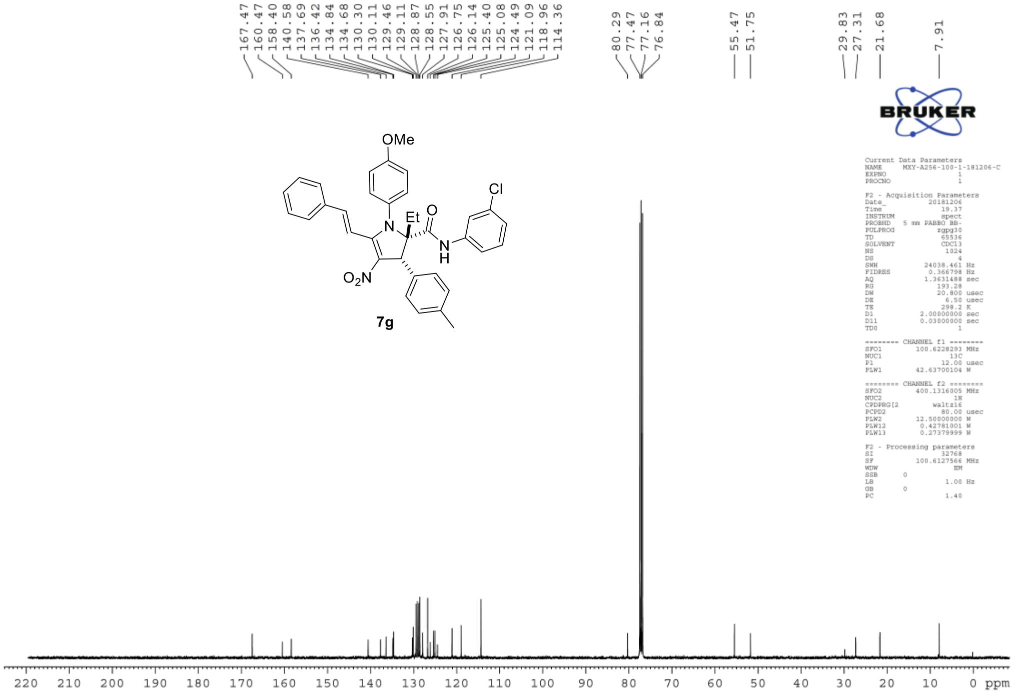
167.47
160.47
158.40
140.58
137.69
136.42
134.84
134.68
130.30
130.11
129.46
129.11
128.87
128.55
127.91
126.75
126.14
125.40
125.08
124.49
121.09
118.96
114.36
80.29
77.47
77.16
76.84
55.47
51.75
29.83
27.31
21.68
7.91
BRUKER
OMe
Cl
N
Et
O
H
$O_2N$
7g
ppm

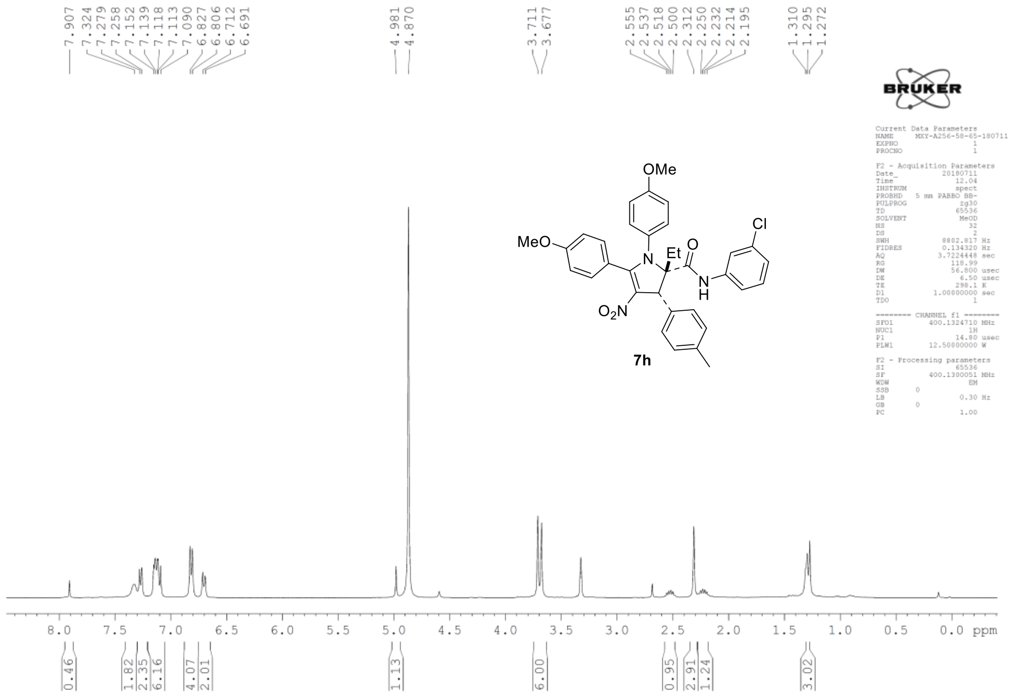

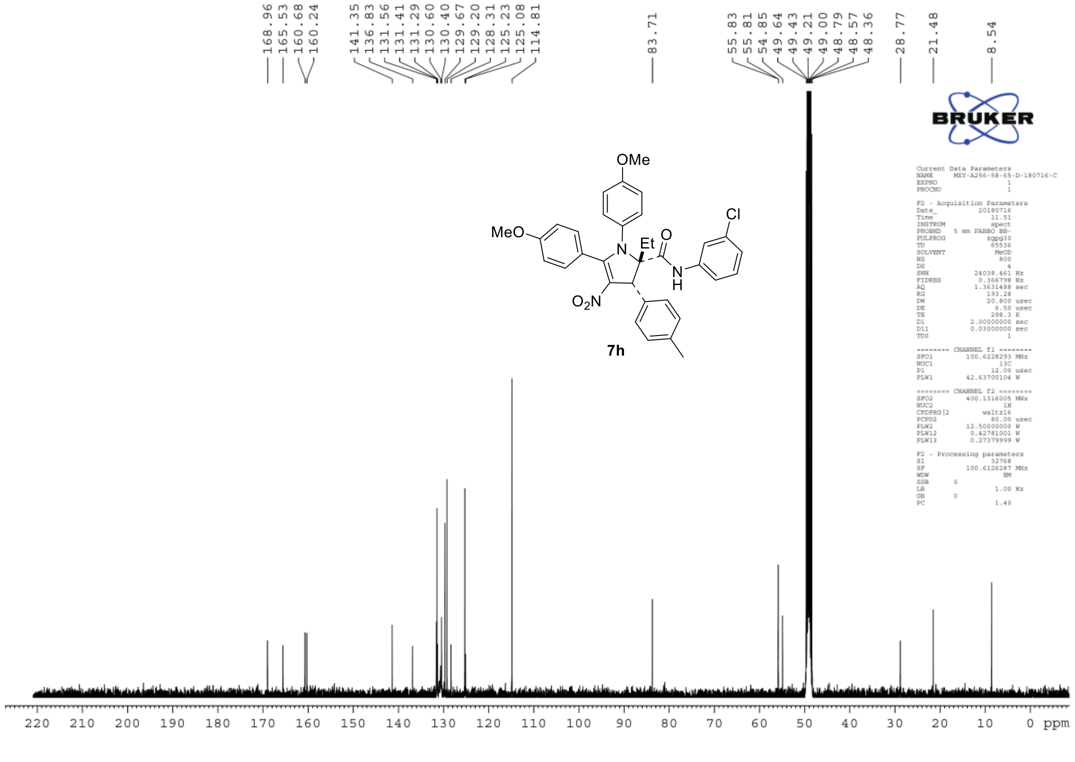

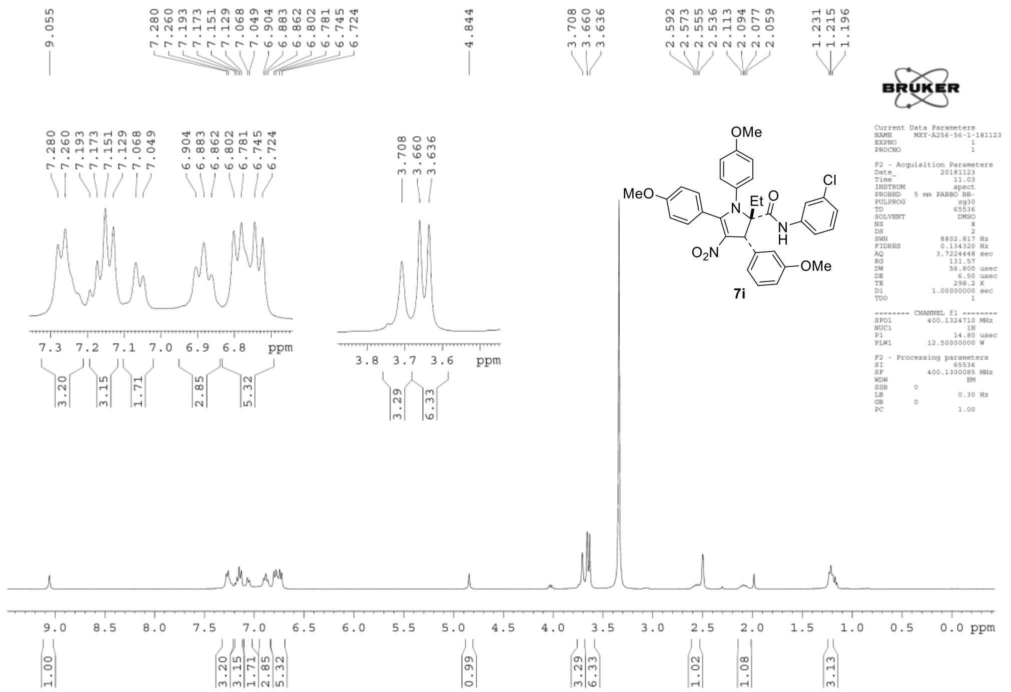
7i
MeO
OMe
Cl
Et
O2N
ppm

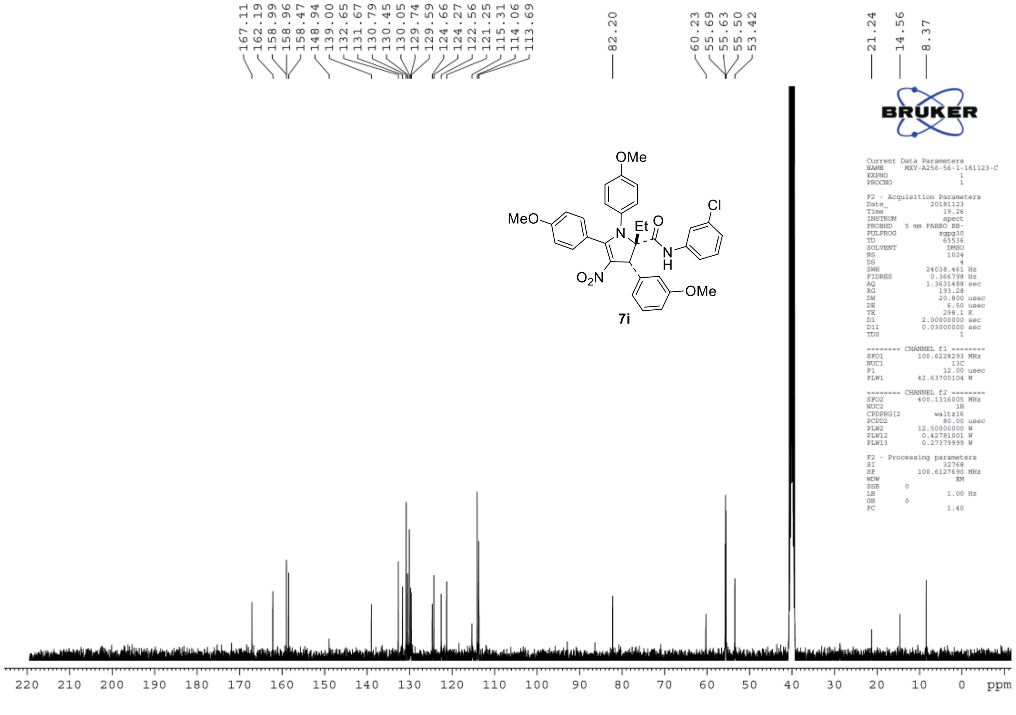
7i
MeO
OMe
Cl
Et
O2N
ppm

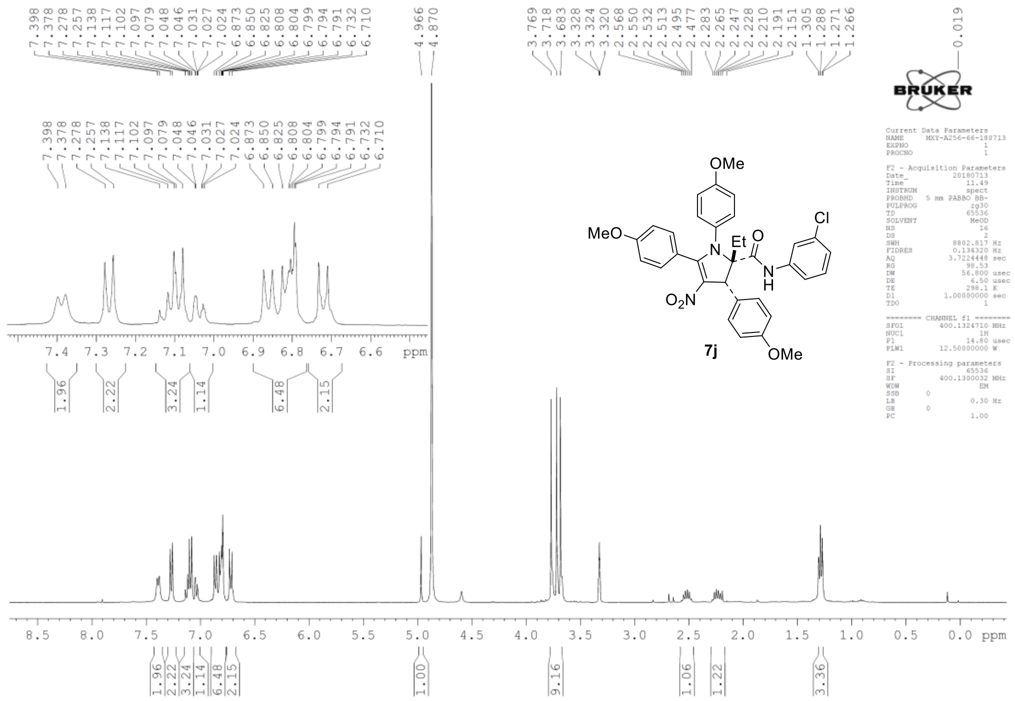

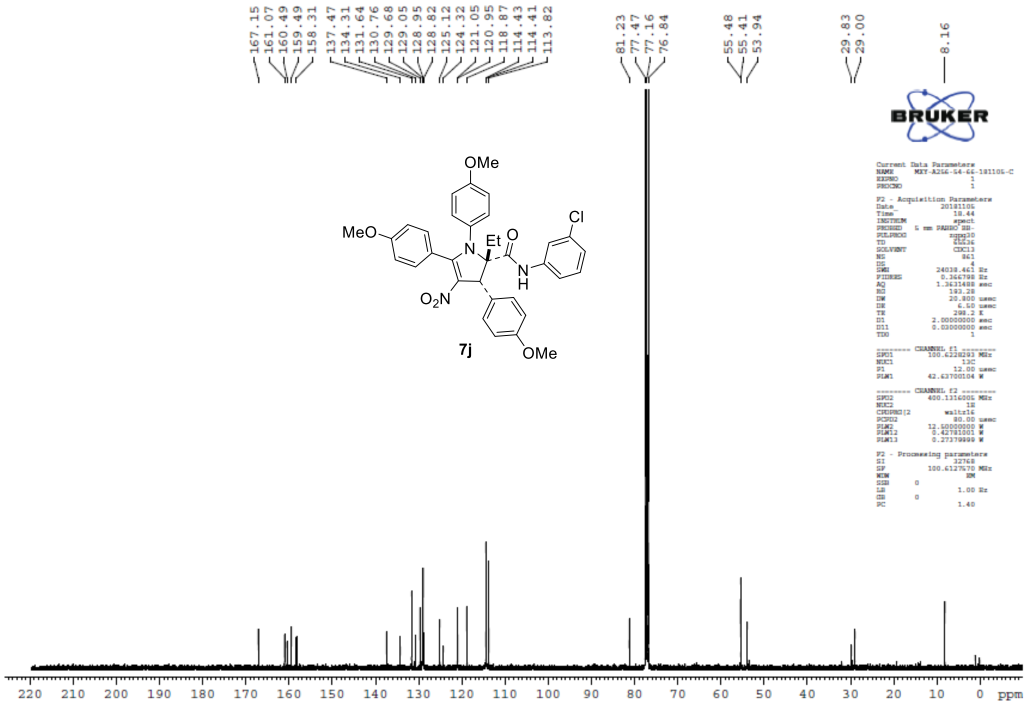

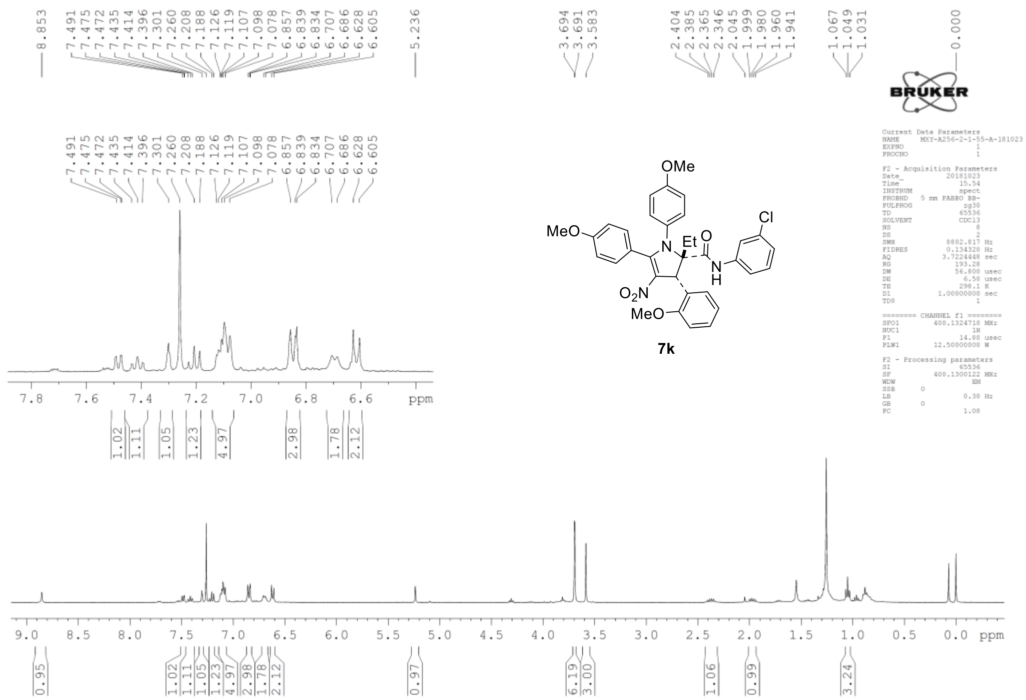

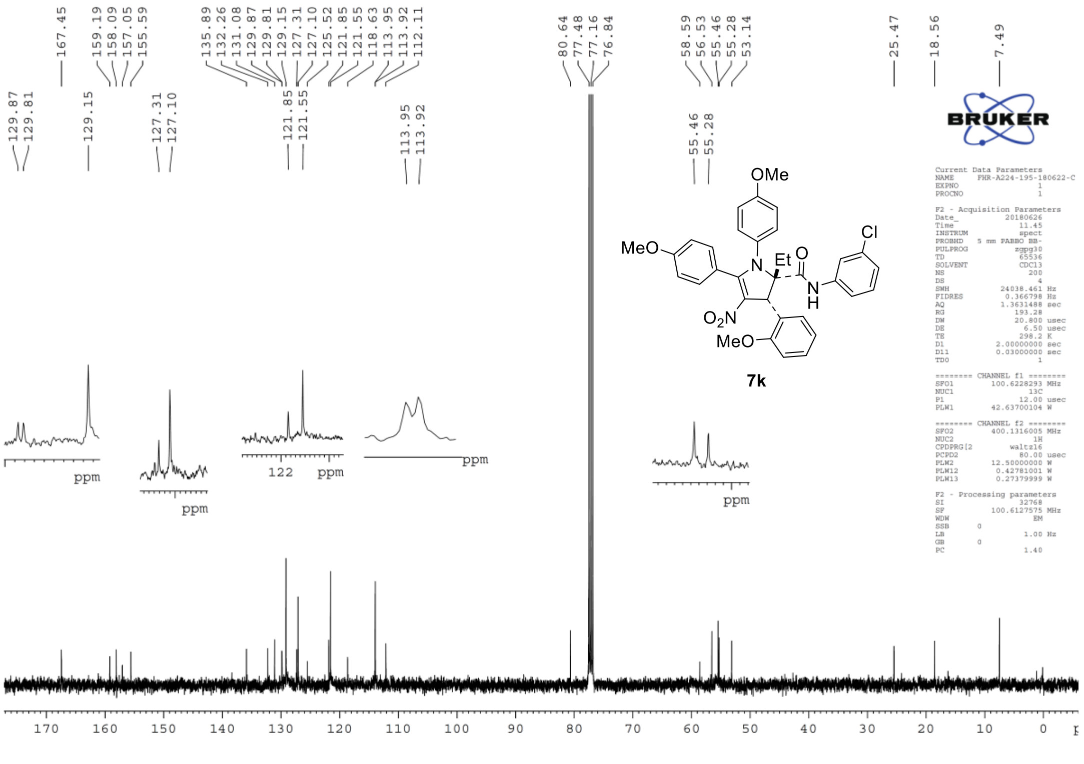

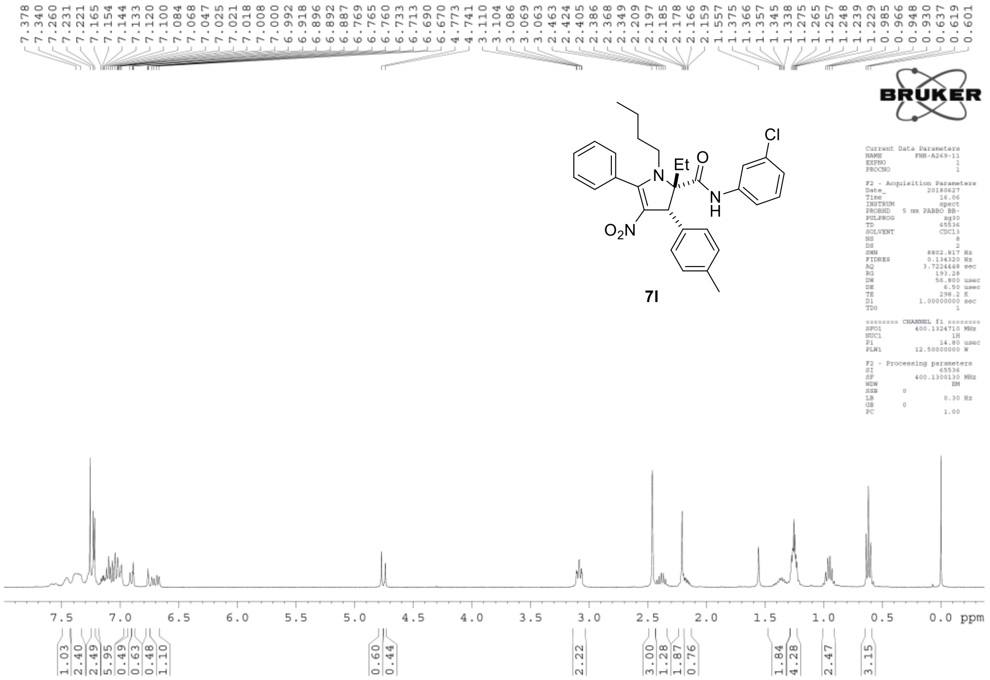

7l


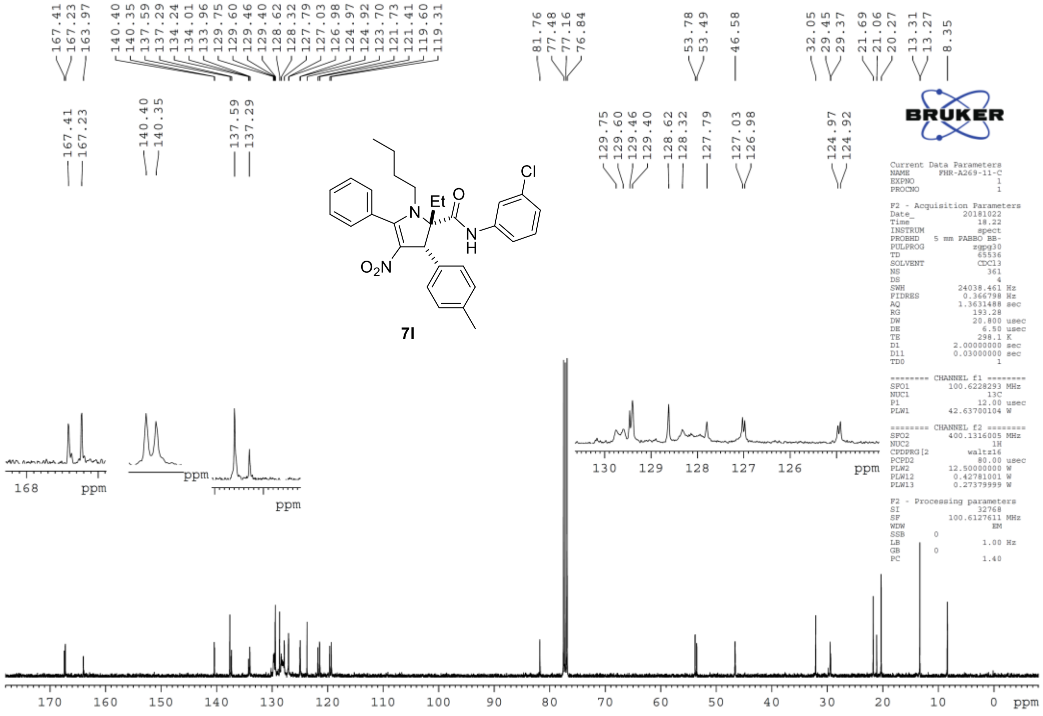

7l

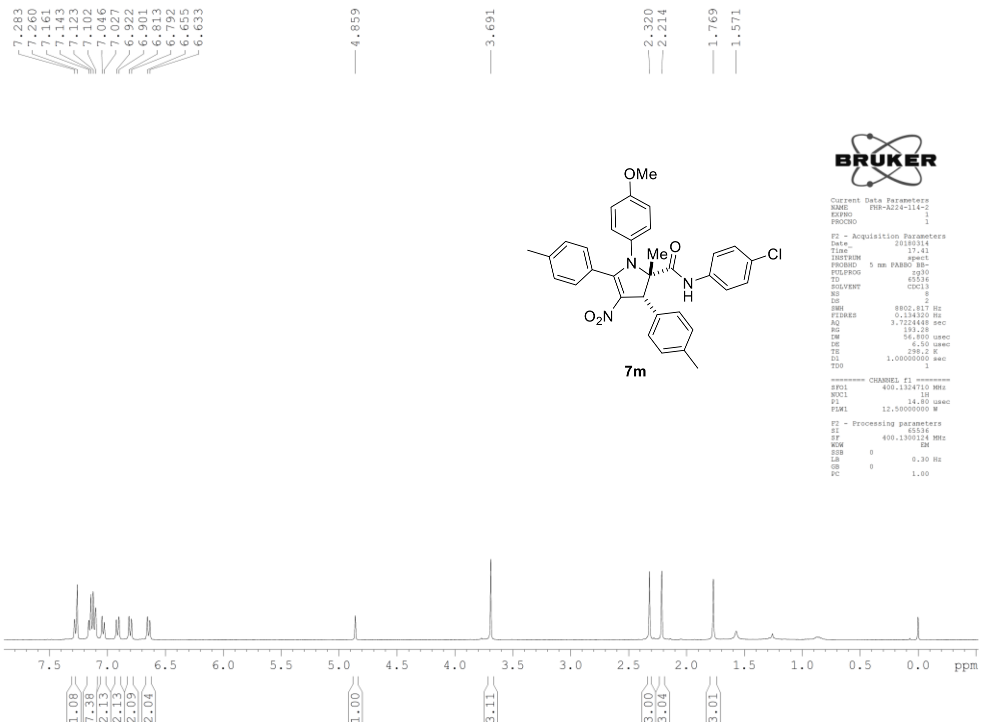
7.283 7.260 7.161 7.143 7.123 7.102 7.046 7.027 6.922 6.901 6.813 6.792 6.655 6.633
4.859
3.691
2.320 2.214
1.769 1.571
OMe
Me
O
N
H
Cl
$O_2N$
7m
BRUKER
Current Data Parameters
F2 - Acquisition Parameters
F2 - Processing parameters
1.08 7.38 2.13 2.13 2.09 2.04 1.00 3.11 3.00 3.04 3.01
ppm

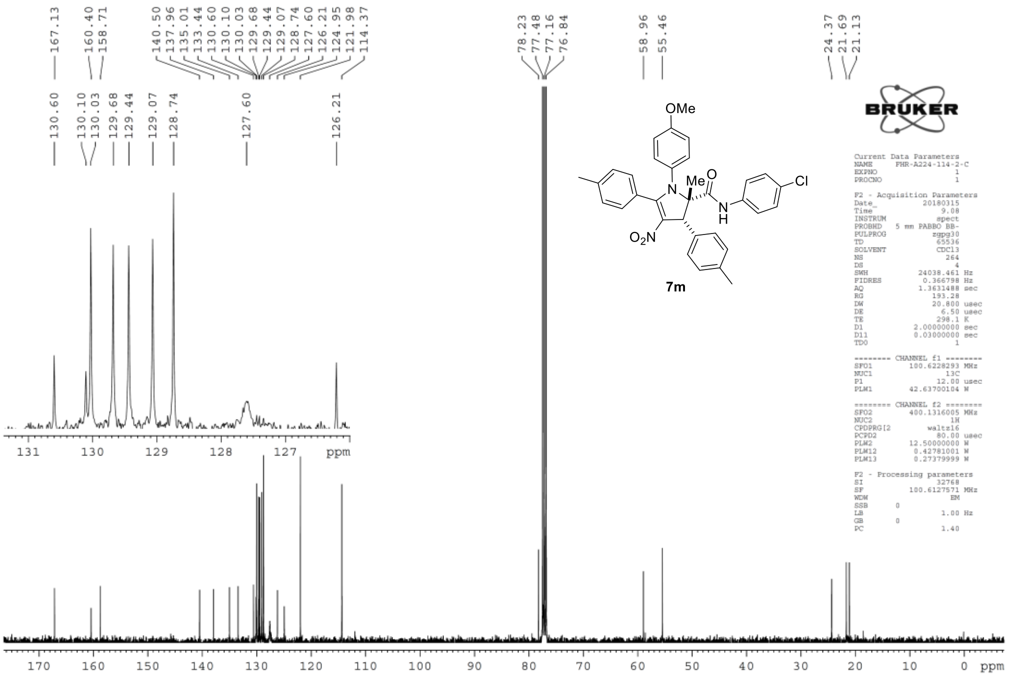
167.13 160.40 158.71 140.50 137.96 135.01 133.44 130.60 130.10 130.03 129.68 129.44 129.07 128.74 127.60 126.21 124.95 121.98 114.37
78.23 77.48 77.16 76.84
58.96 55.46
24.37 21.69 21.13
130.60 130.10 130.03 129.68 129.44 129.07 128.74 127.60 126.21
OMe
Me
O
N
H
Cl
$O_2N$
7m
BRUKER
Current Data Parameters
F2 - Acquisition Parameters
F2 - Processing parameters
ppm

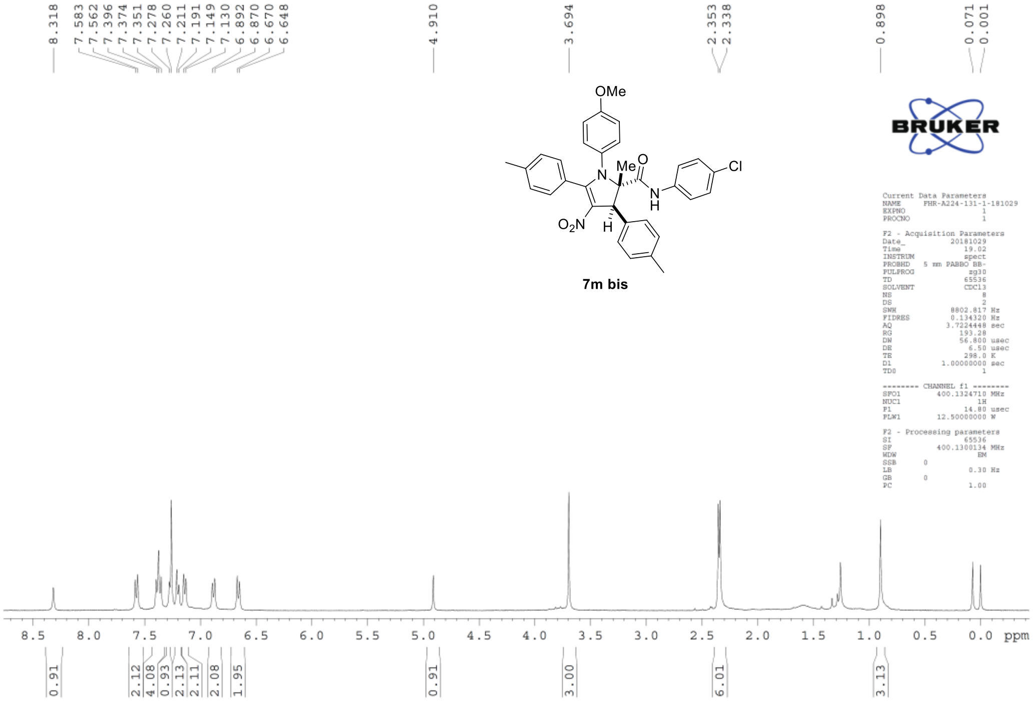

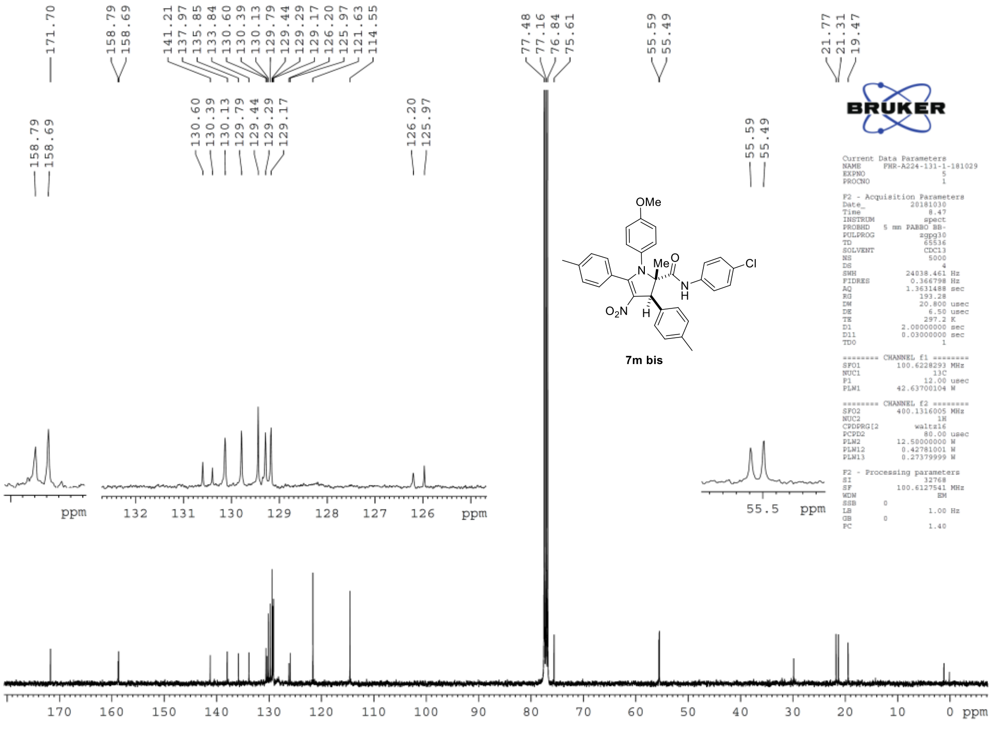

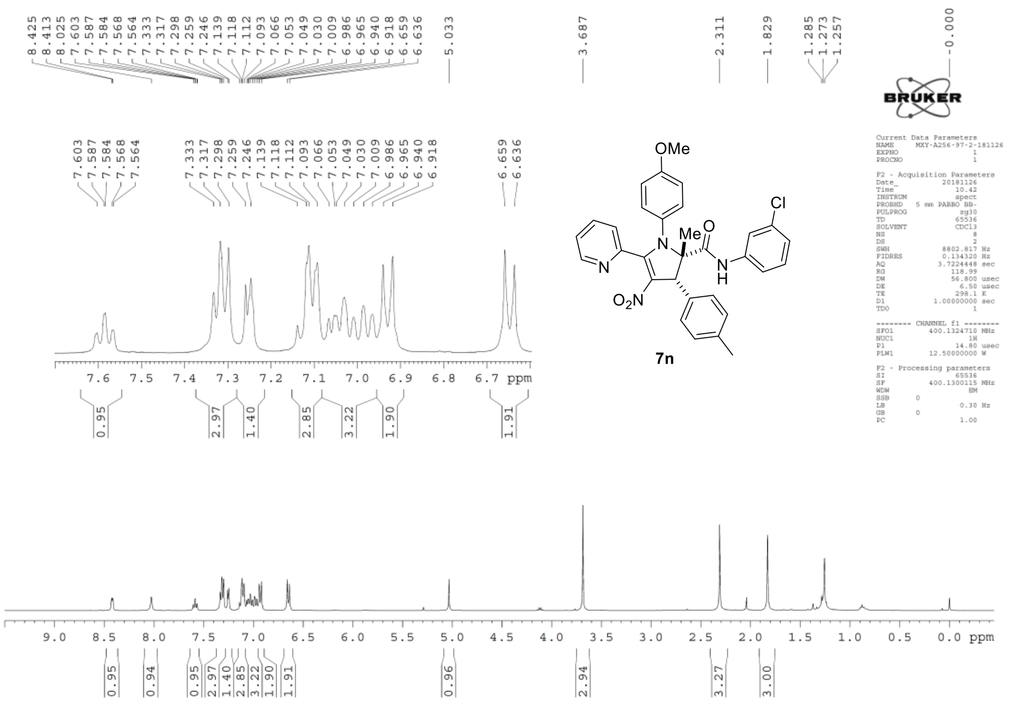

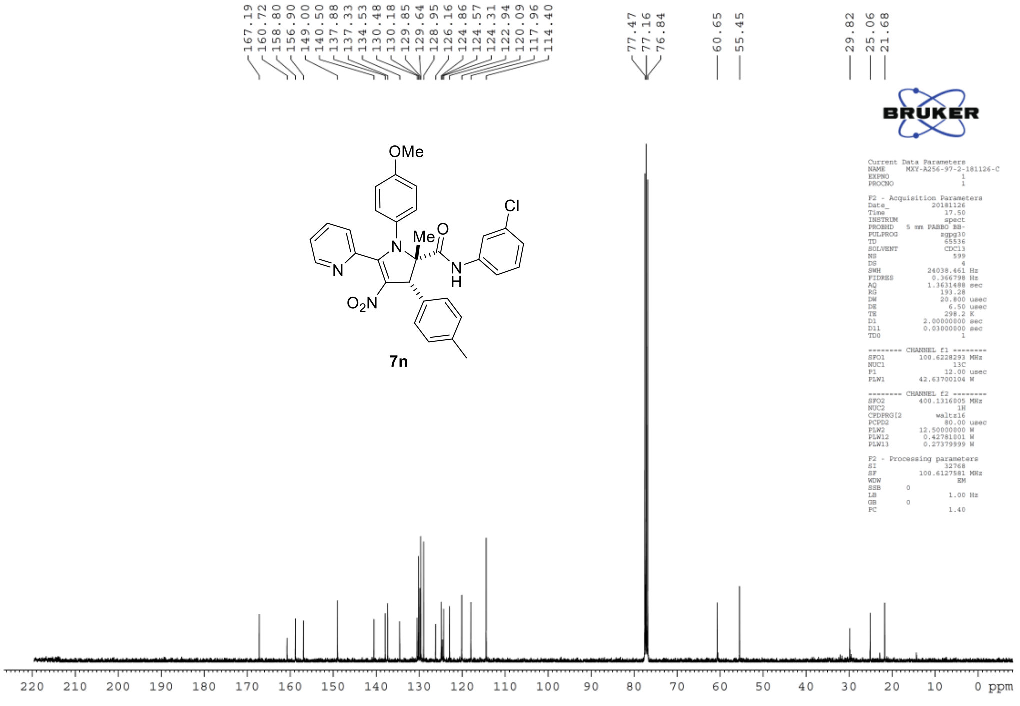

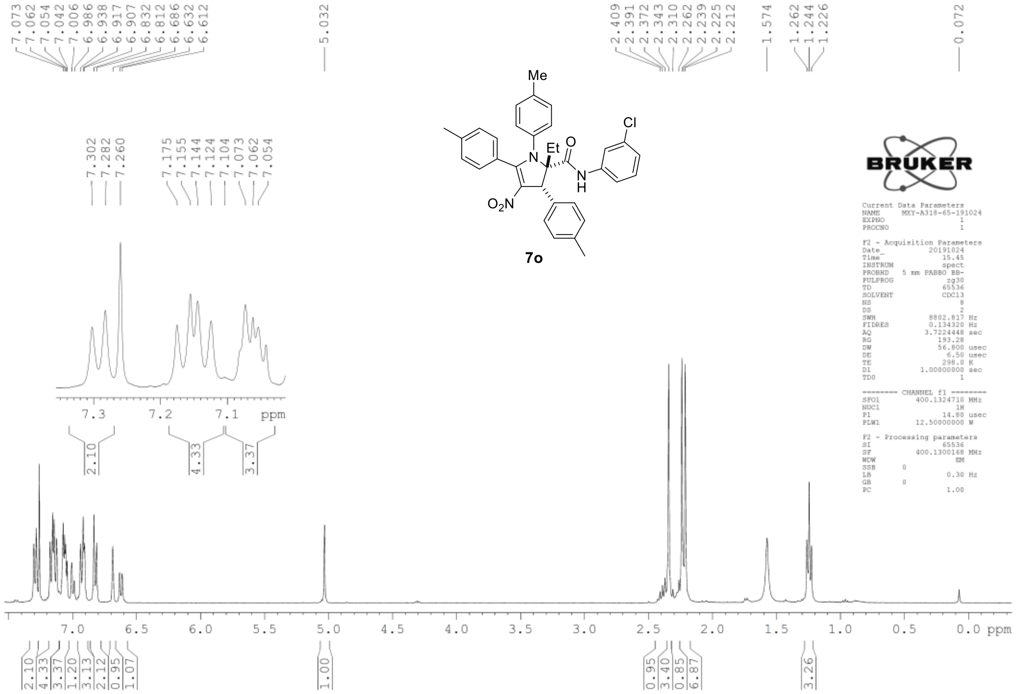
7.302
7.282
7.260
7.175
7.155
7.144
7.124
7.104
7.073
7.062
7.054
7.042
7.006
6.986
6.938
6.917
6.907
6.832
6.812
6.686
6.632
6.612
5.032
2.409
2.391
2.372
2.343
2.310
2.262
2.239
2.225
2.212
1.574
1.262
1.244
1.226
0.072
Me
Cl
Et
O
N
H
O2N
7o
BRUKER
ppm

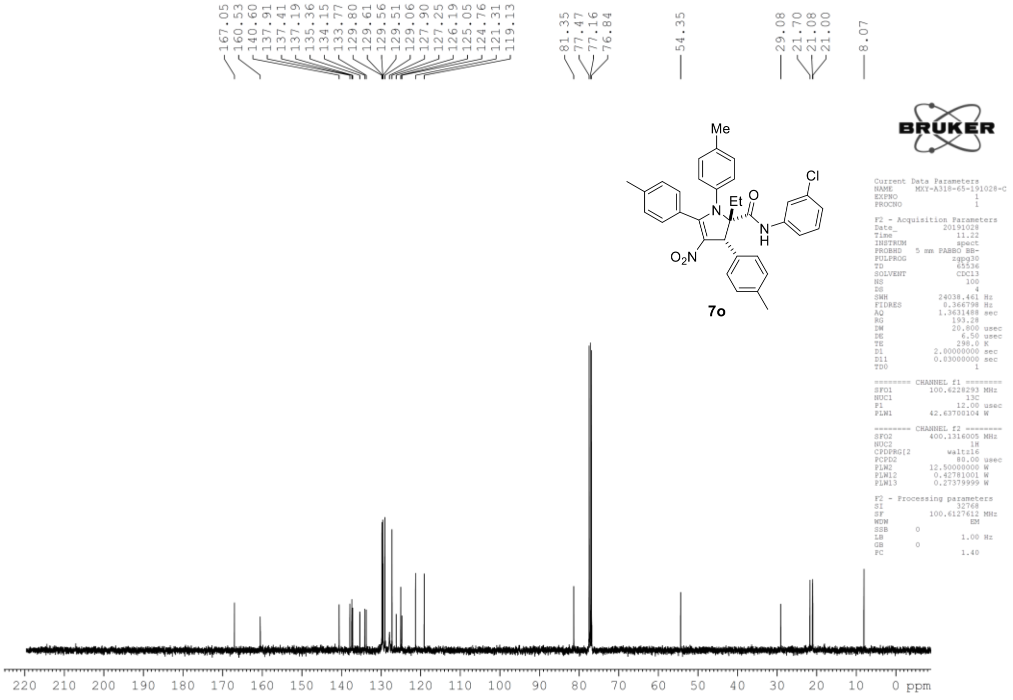
167.05
160.53
140.60
137.91
137.41
137.19
135.36
134.15
133.77
129.80
129.61
129.56
129.51
129.06
127.90
127.25
126.19
125.05
124.76
121.31
119.13
81.35
77.47
77.16
76.84
54.35
29.08
21.70
21.08
21.00
8.07
Me
Cl
Et
O
N
H
O2N
7o
BRUKER
ppm

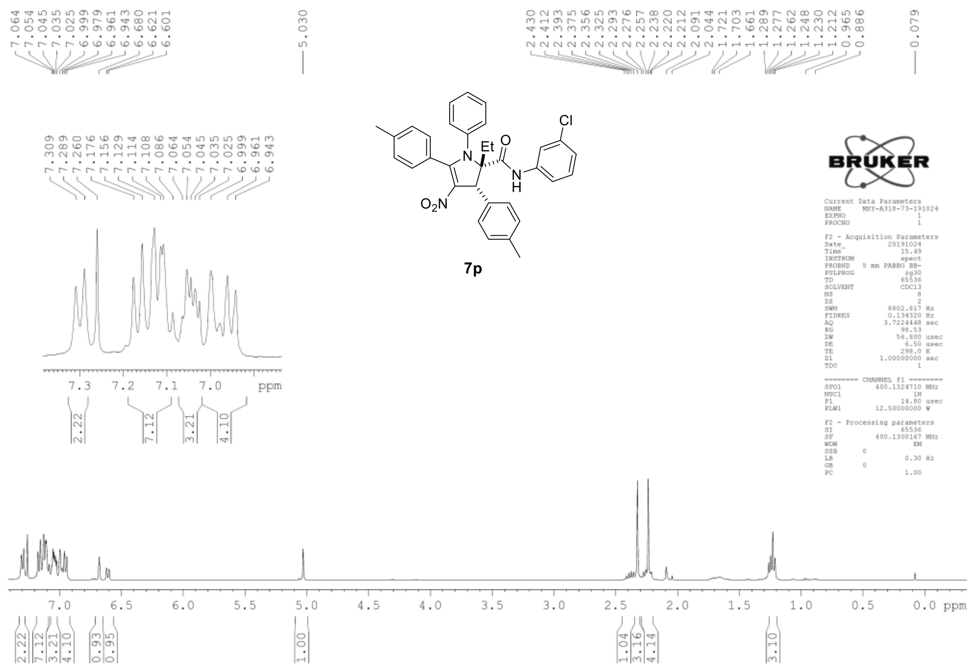

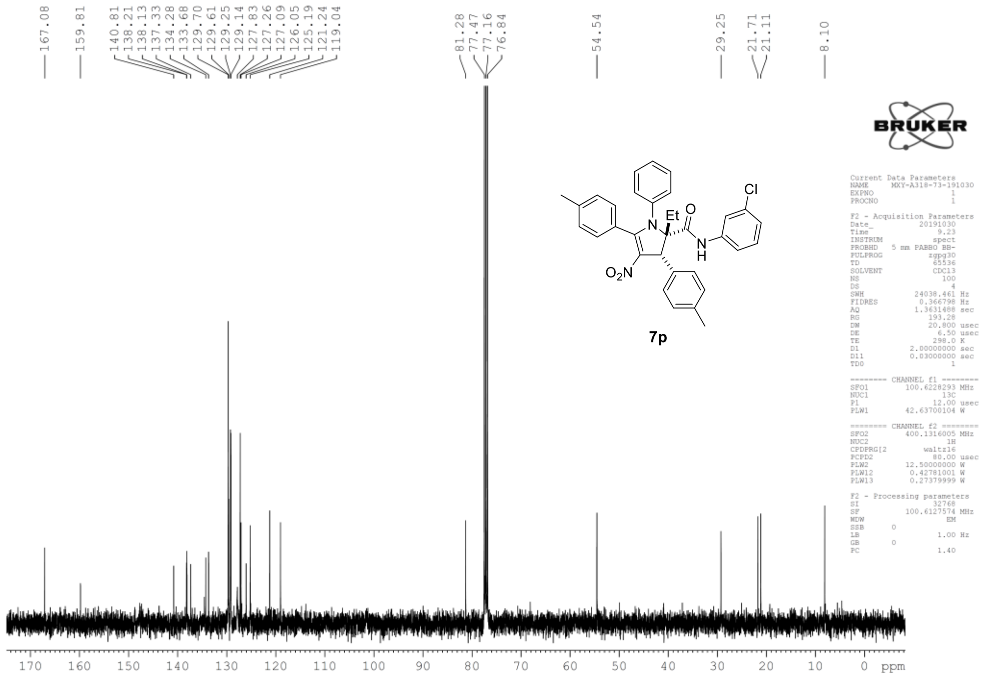

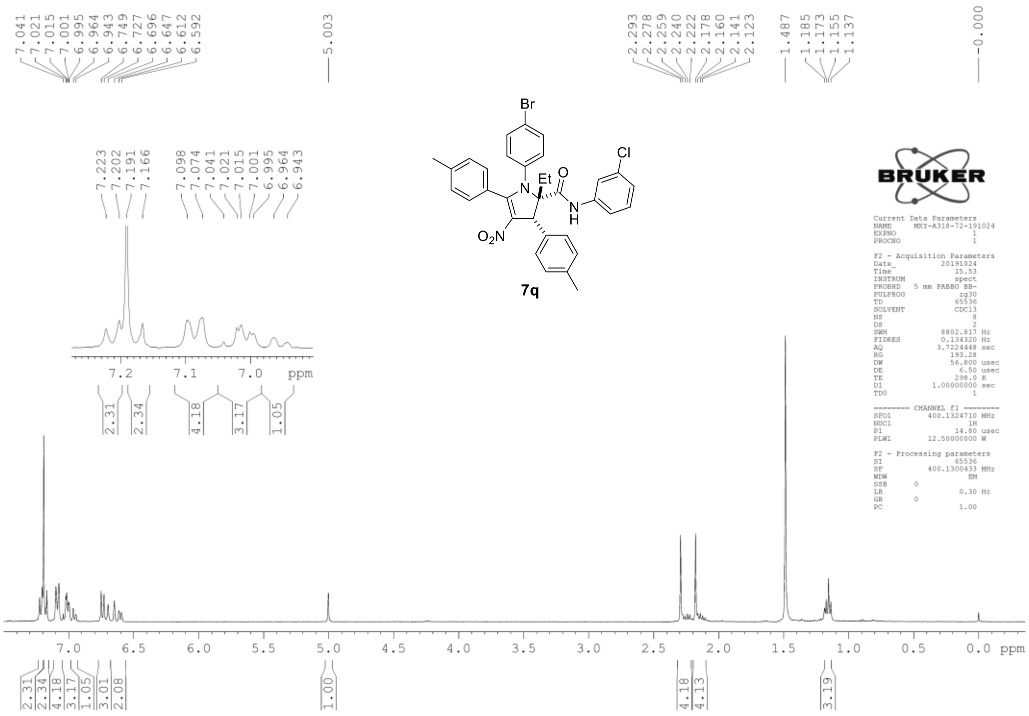
7.041 7.021 7.015 7.001 6.995 6.964 6.943 6.749 6.727 6.696 6.647 6.612 6.592
5.003
2.293 2.278 2.259 2.240 2.222 2.178 2.160 2.141 2.123
1.487 1.185 1.173 1.155 1.137
-0.000
7.223 7.202 7.191 7.166 7.098 7.074 7.041 7.021 7.015 7.001 6.995 6.964 6.943
Br
Cl
Et
O
N
H
$O_2N$
7q
BRUKER
ppm

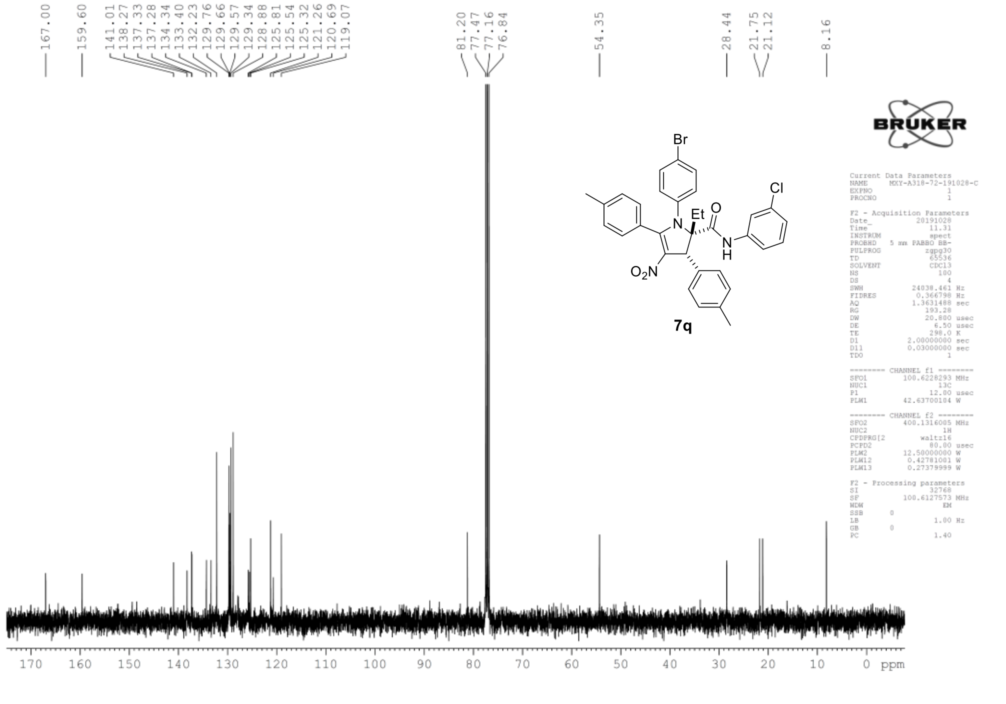
167.00
159.60
141.01 138.27 137.33 137.28 134.34 133.40 132.23 129.76 129.66 129.57 129.34 128.88 125.81 125.54 125.32 121.26 120.69 119.07
81.20 77.47 77.16 76.84
54.35
28.44 21.75 21.12
8.16
Br
Cl
Et
O
N
H
$O_2N$
7q
BRUKER
ppm

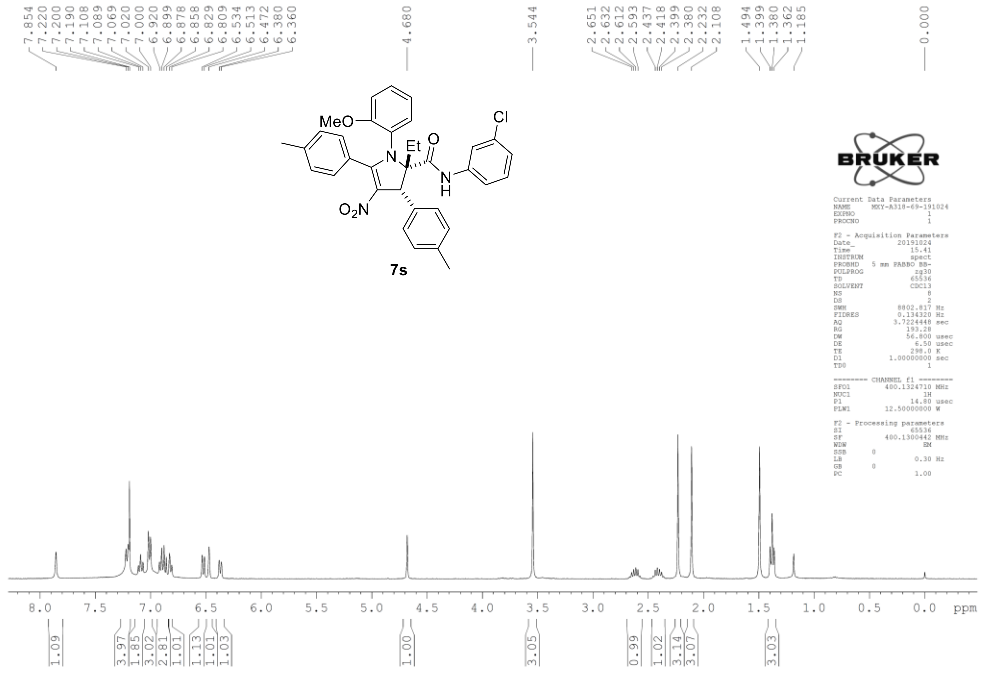
7.854 7.220 7.200 7.190 7.108 7.089 7.069 7.020 7.000 6.920 6.899 6.878 6.858 6.829 6.809 6.534 6.513 6.472 6.380 6.360
4.680
3.544
2.651 2.632 2.612 2.593 2.437 2.418 2.399 2.380 2.232 2.108
1.494 1.399 1.380 1.362 1.185
0.000
MeO
Et
O
Cl
N
H
$O_2N$
7s
BRUKER
1.09 3.97 1.85 3.02 2.81 1.01 1.13 1.01 1.03 1.00 3.05 0.99 1.02 3.14 3.07 3.03
ppm

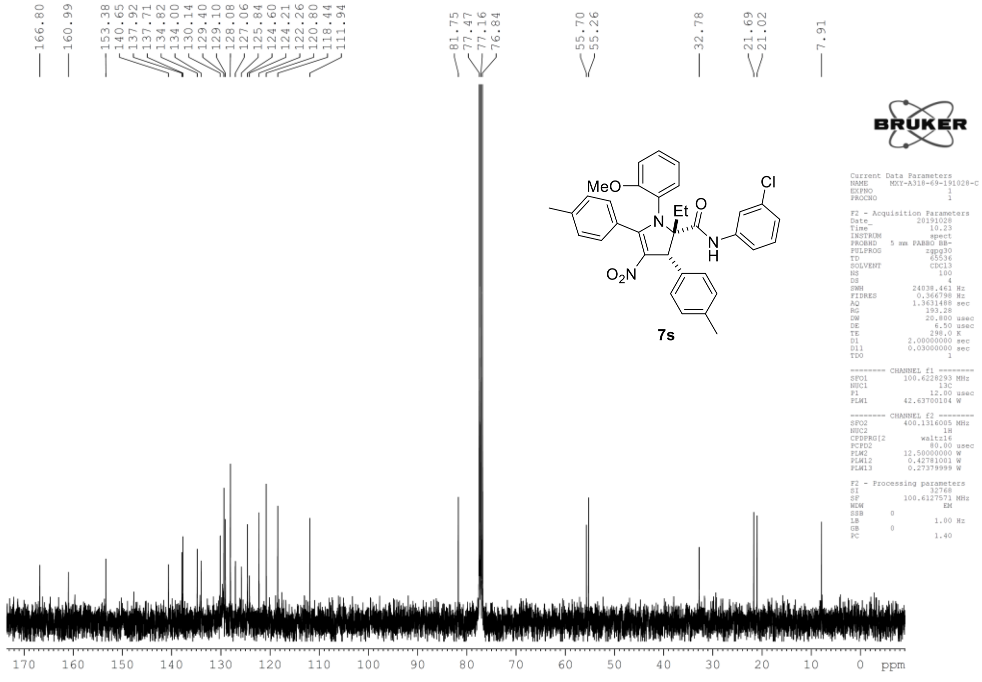
166.80 160.99 153.38 140.65 137.92 137.71 134.82 134.00 130.14 129.40 129.10 128.08 127.06 125.84 124.60 124.21 122.26 120.80 118.44 111.94
81.75 77.47 77.16 76.84
55.70 55.26
32.78
21.69 21.02
7.91
BRUKER
MeO
Et
O
Cl
N
H
$O_2N$
7s
ppm

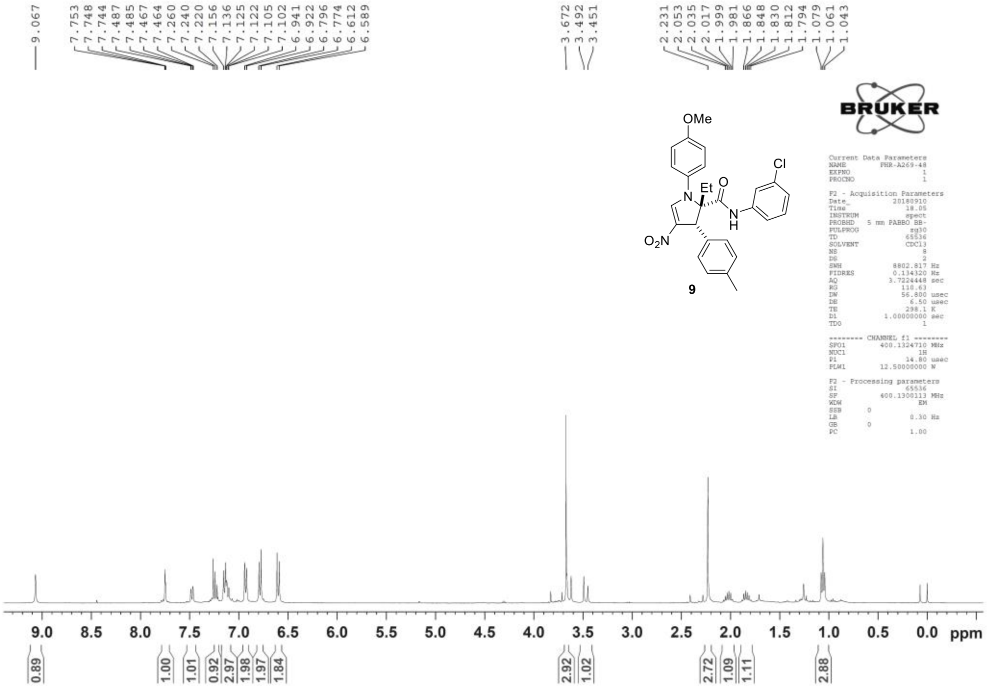
9.067
7.753 7.748 7.744 7.487 7.485 7.467 7.464 7.260 7.240 7.220 7.156 7.136 7.125 7.122 7.105 7.102 6.941 6.922 6.796 6.774 6.612 6.589
3.672 3.492 3.451
2.231 2.053 2.035 2.017 1.999 1.981 1.866 1.848 1.830 1.812 1.794 1.079 1.061 1.043
BRUKER
OMe
Cl
Et
O
N
H
$O_2N$
9
9.0 8.5 8.0 7.5 7.0 6.5 6.0 5.5 5.0 4.5 4.0 3.5 3.0 2.5 2.0 1.5 1.0 0.5 0.0 ppm
0.89 1.00 1.01 0.92 2.97 1.98 1.97 1.84 2.92 1.02 2.72 1.09 1.11 2.88

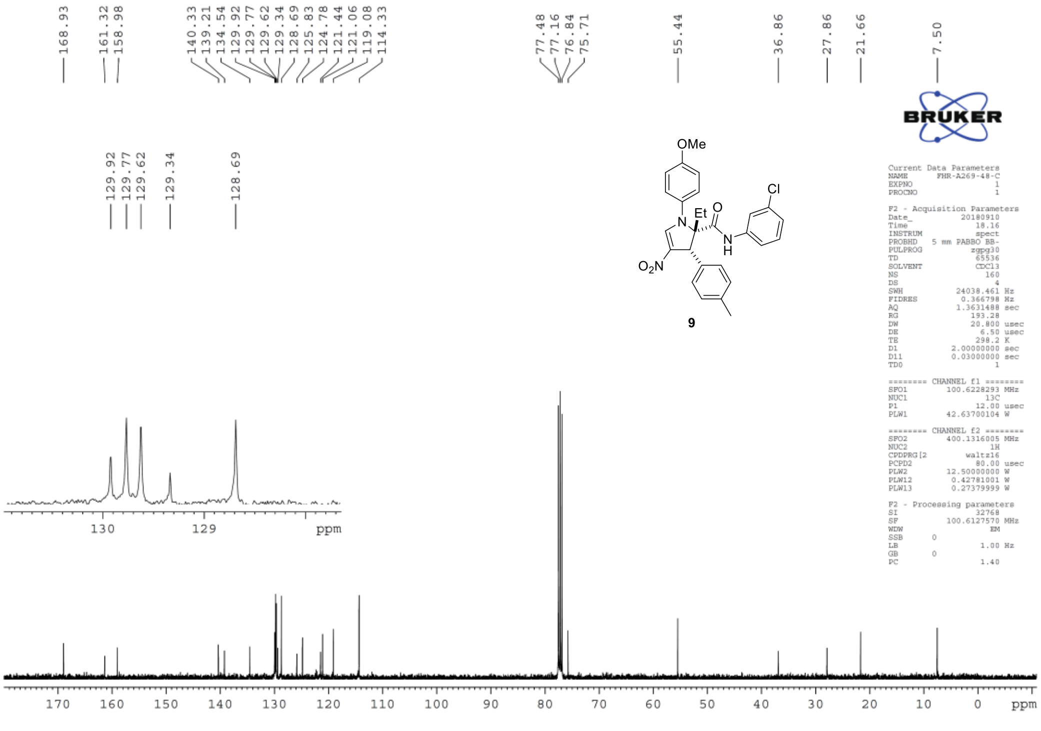
168.93
161.32 158.98
140.33 139.21 134.54 129.92 129.77 129.62 129.34 128.69 125.83 124.78 121.44 121.06 119.08 114.33
77.48 77.16 76.84 75.71
55.44
36.86
27.86
21.66
7.50
BRUKER
129.92 129.77 129.62 129.34 128.69
130 129 ppm
OMe
Cl
Et
O
N
H
$O_2N$
9
170 160 150 140 130 120 110 100 90 80 70 60 50 40 30 20 10 0 ppm

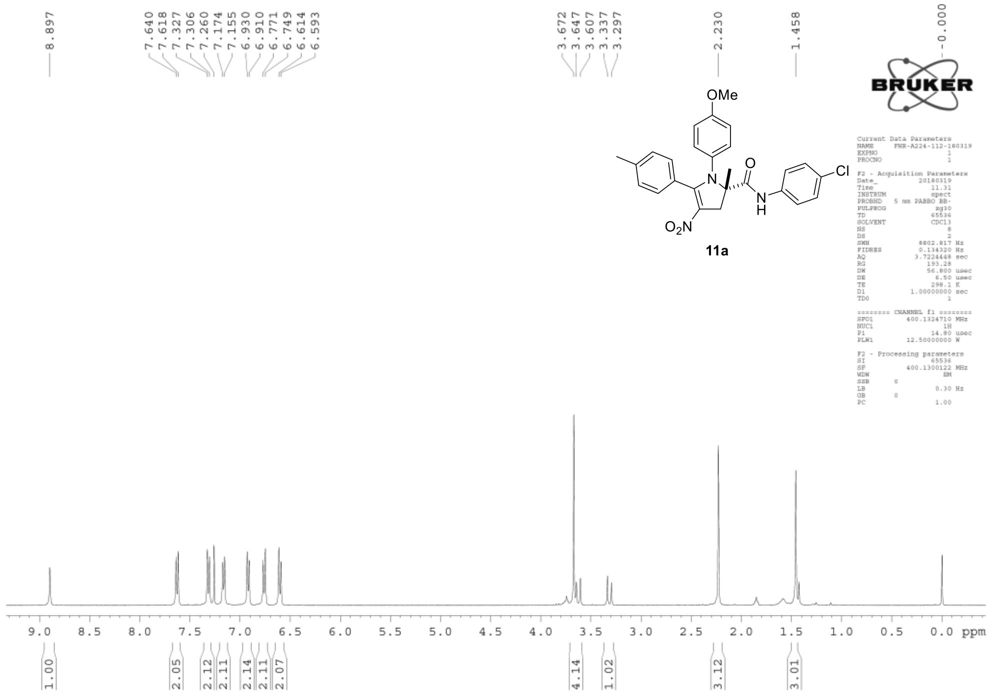

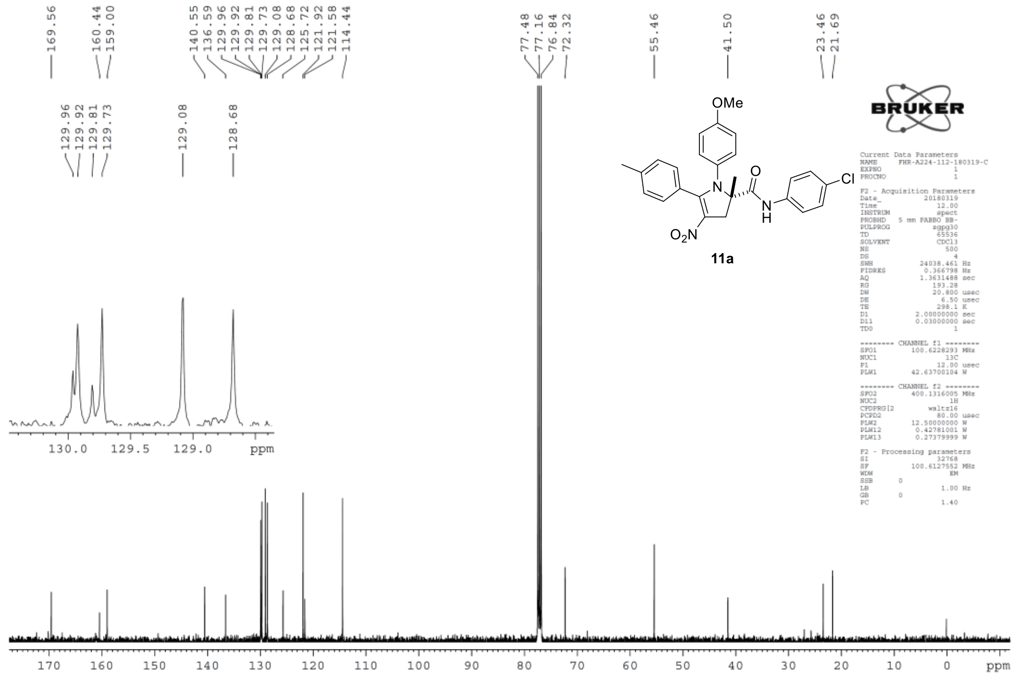

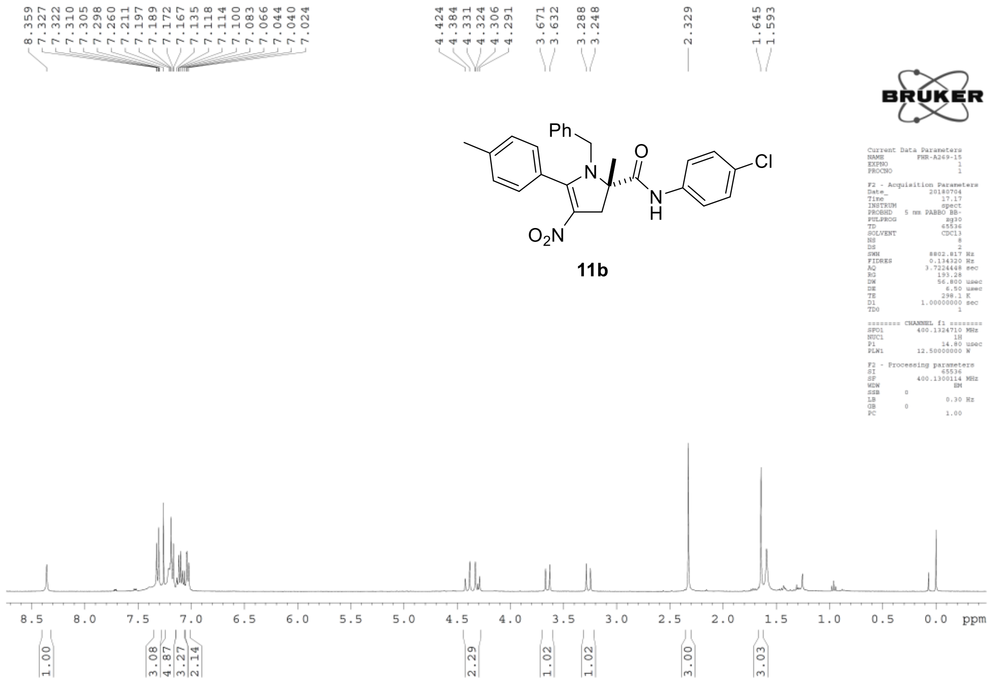
11b

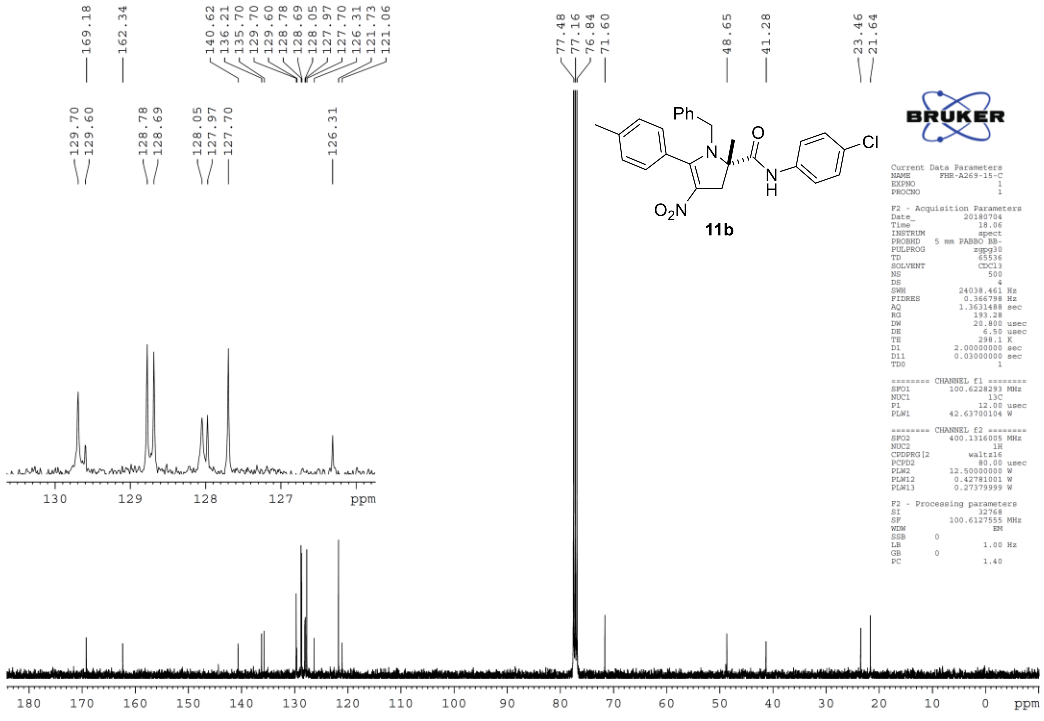
11b

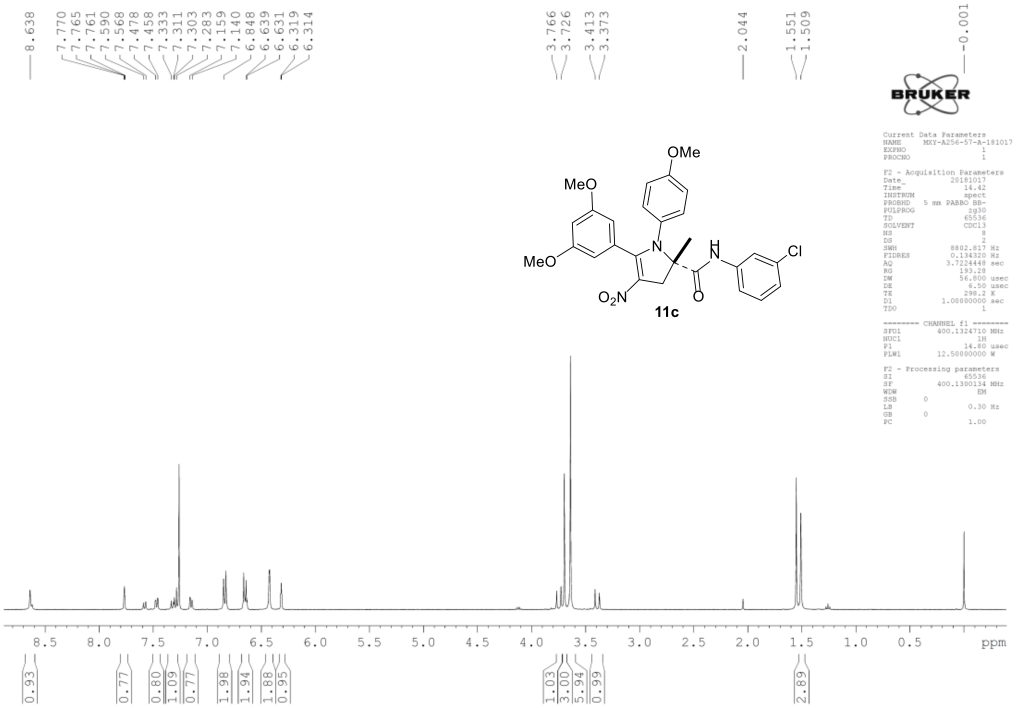

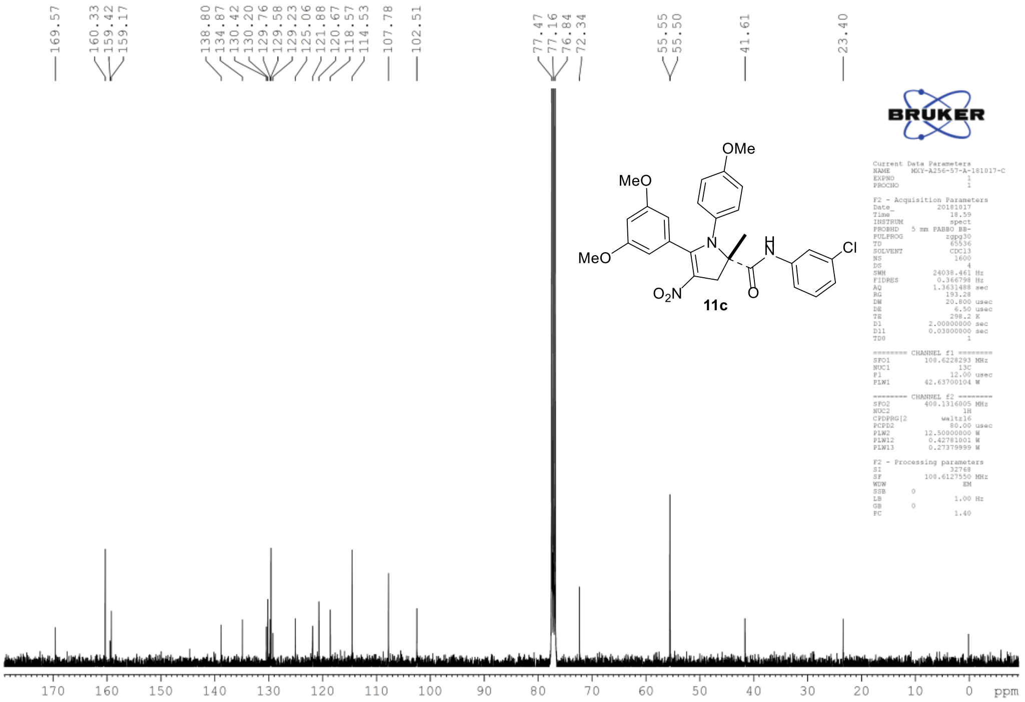

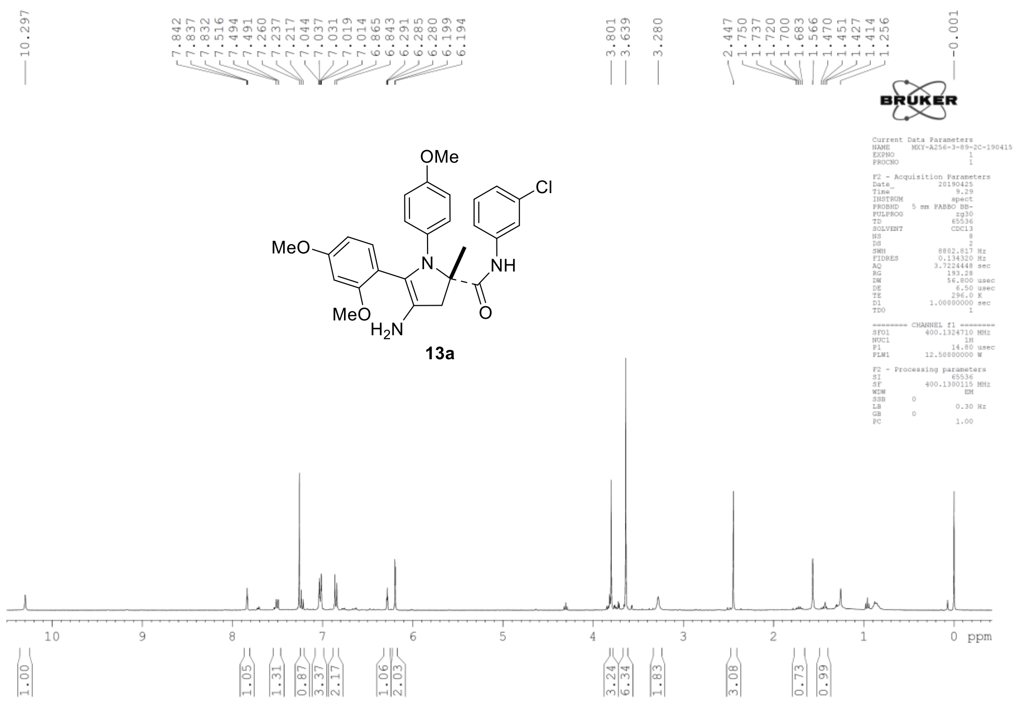

10.297
7.842 7.837 7.832 7.516 7.494 7.491 7.260 7.237 7.217 7.044 7.037 7.031 7.019 7.014 6.865 6.843 6.291 6.285 6.280 6.199 6.194
3.801 3.639 3.280
2.447 1.750 1.737 1.720 1.700 1.683 1.566 1.470 1.451 1.427 1.414 1.256
-0.001
BRUKER
OMe
Cl
MeO
N
NH
MeO
$H_2N$
O
13a
ppm
1.00 1.05 1.31 0.87 3.37 2.17 1.06 2.03 3.24 6.34 1.83 3.08 0.73 0.99


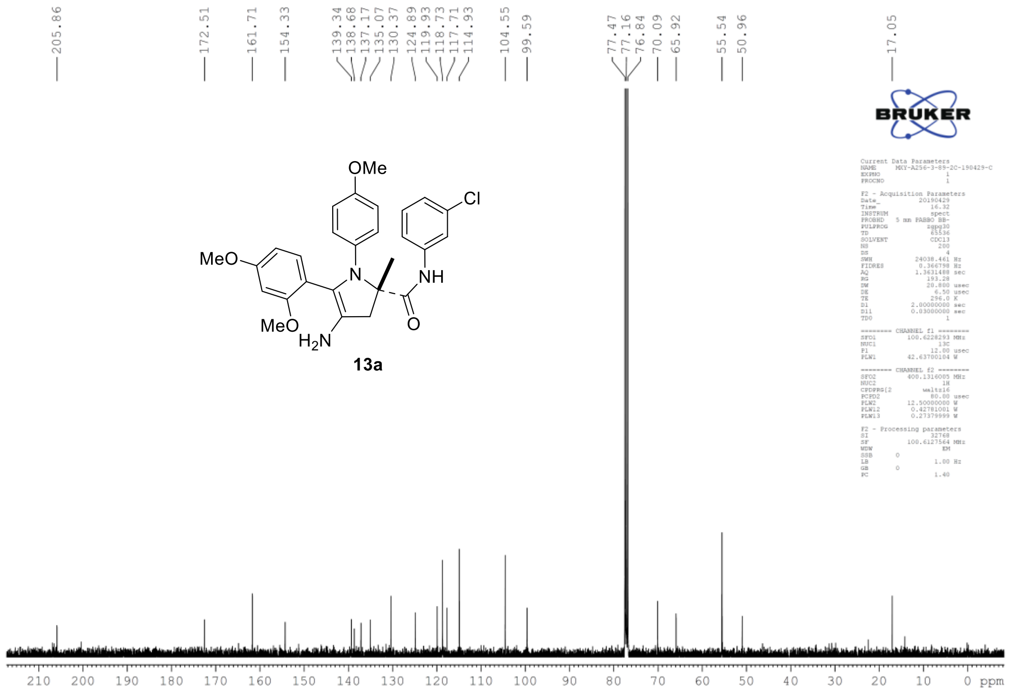

205.86
172.51 161.71 154.33
139.34 138.68 137.17 135.07 130.37 124.89 119.93 118.73 117.71 114.93
104.55 99.59
77.47 77.16 76.84 70.09 65.92
55.54 50.96
17.05
BRUKER
OMe
Cl
MeO
N
NH
MeO
$H_2N$
O
13a
ppm

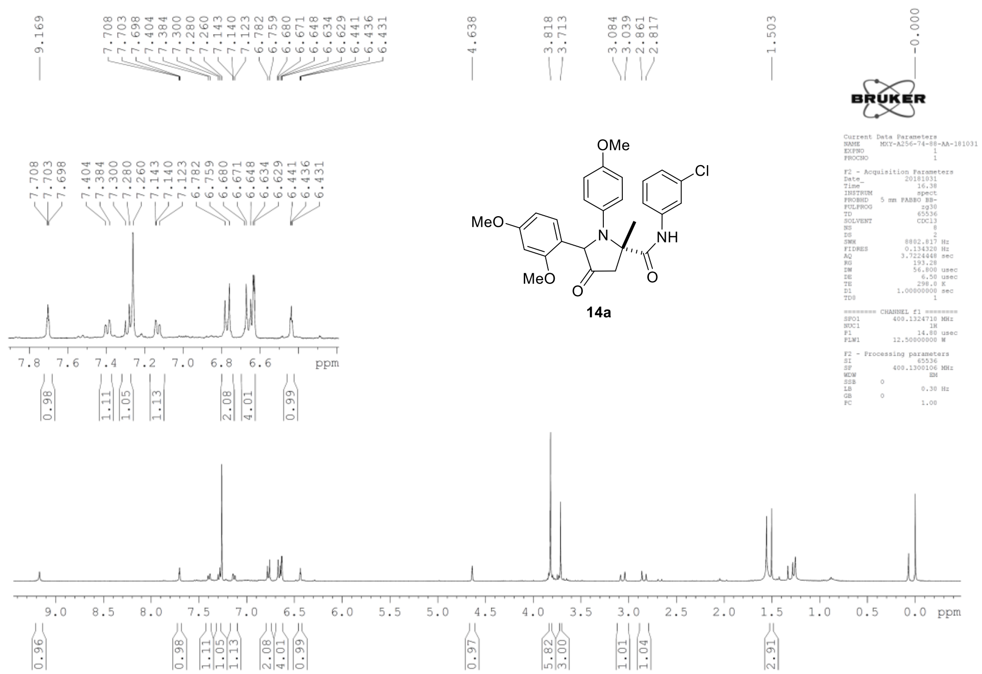
9.169
7.708 7.703 7.698 7.404 7.384 7.300 7.280 7.260 7.143 7.140 7.123 6.782 6.759 6.680 6.671 6.648 6.634 6.629 6.441 6.436 6.431
4.638
3.818 3.713
3.084 3.039 2.861 2.817
1.503
0.000
BRUKER
OMe
Cl
MeO
N
NH
MeO
O
O
14a
ppm

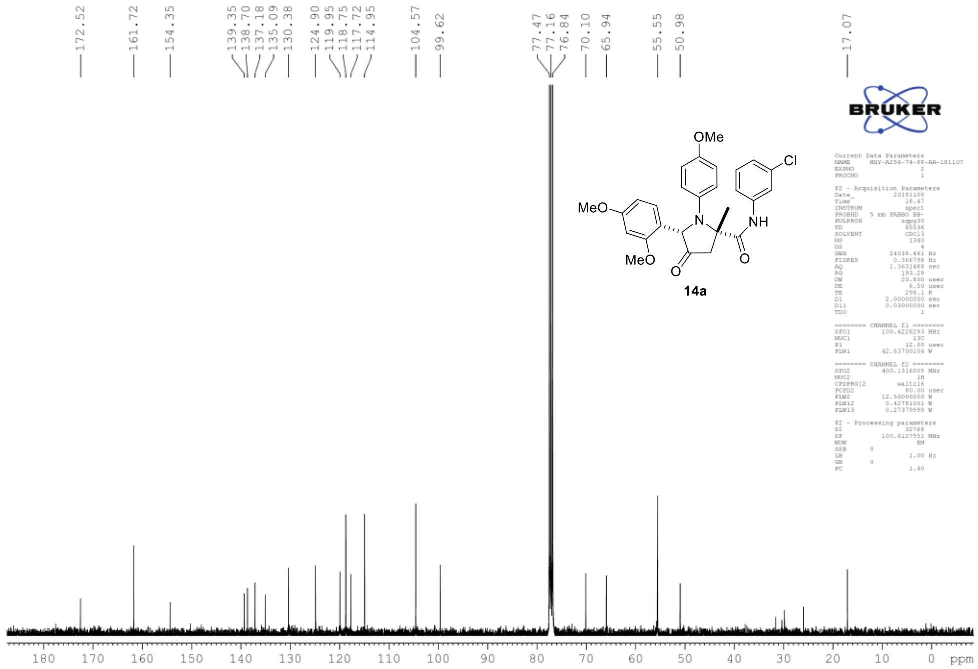
172.52
161.72
154.35
139.35 138.70 137.18 135.09 130.38
124.90 119.95 118.75 117.72 114.95
104.57 99.62
77.47 77.16 76.84
70.10 65.94
55.55 50.98
17.07
BRUKER
OMe
Cl
MeO
N
NH
MeO
O
O
14a
ppm

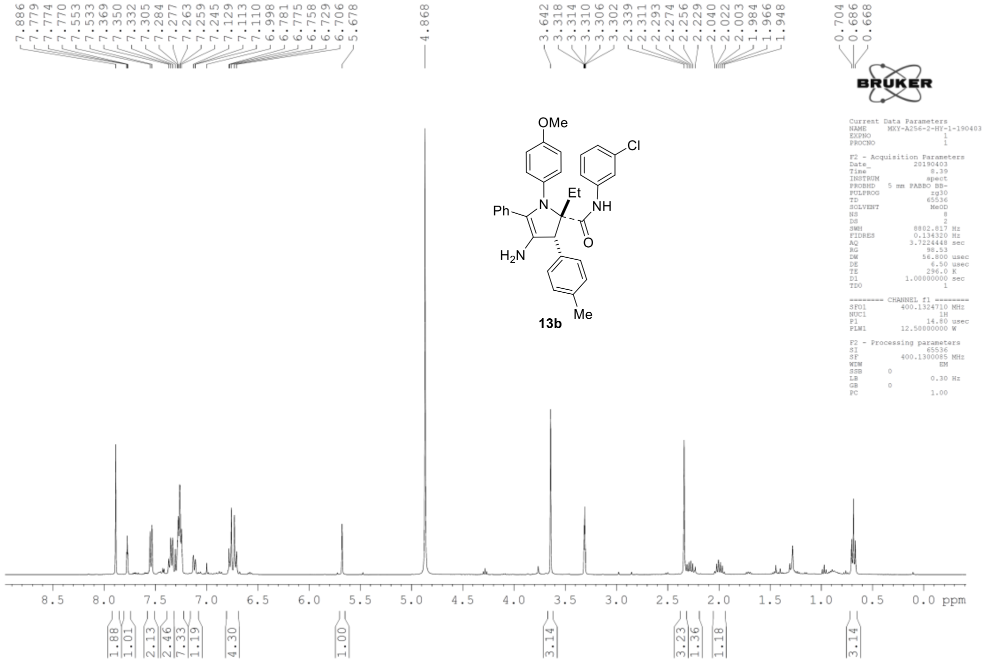
13b
OMe
Cl
Et
NH
Ph
N
O
H2N
Me
BRUKER

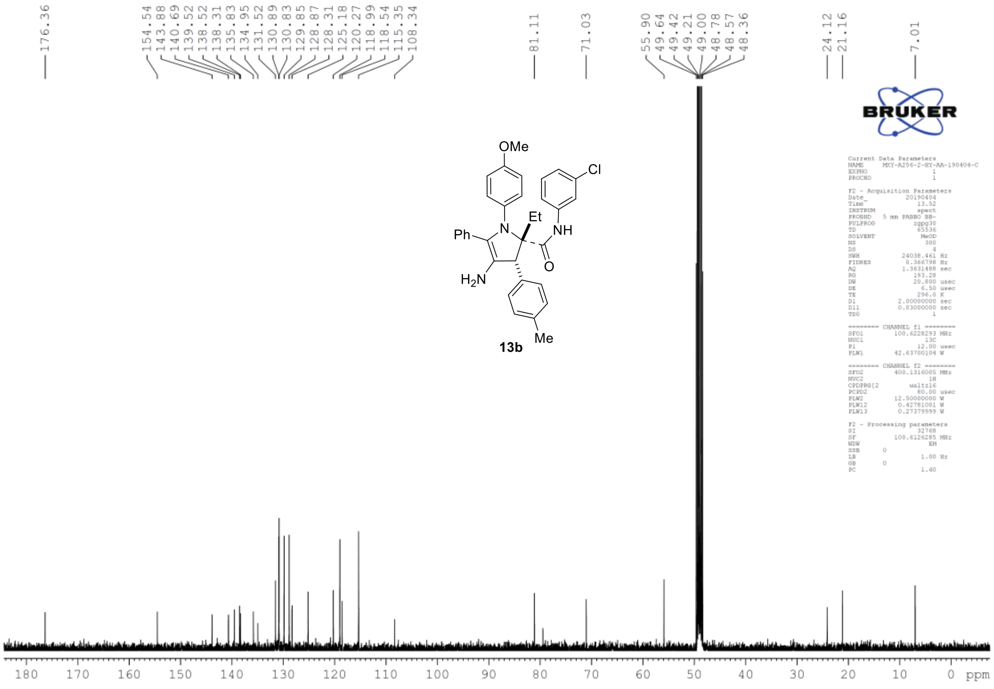
13b
OMe
Cl
Et
NH
Ph
N
O
H2N
Me
BRUKER
